# Surface enhanced Raman spectroscopy of *Chlamydia trachomatis* and *Neisseria gonorrhoeae* for diagnostics, and extra-cellular metabolomics and biochemical monitoring

**DOI:** 10.1038/s41598-018-23562-5

**Published:** 2018-03-26

**Authors:** Y. Chen, W. R. Premasiri, L. D. Ziegler

**Affiliations:** Department of Chemistry and The Photonics Center, Boston University, Boston, MA 02215 USA

## Abstract

SERS spectra excited at 785 nm of the bacteria *Chlamydia trahomatis* (elementary bodies, EB) and *Neisseria gonorrheoae*, the causative pathogens for the two most common sexually transmitted diseases (STD), chlamydia and gonorrhea, respectively, are reported. Although both are Gram-negative bacteria, the SERS signatures of *C*. *trachomatis* and *N*. *gonorrheoae* are completely different. *N*. *gonorrheoae* SERS spectra are due to the starvation induced nucleotide metabolites adenine and guanine, and the surface associated co-enzyme nicotinamide adenine dinucleotide and are very similar on Au and Ag although the spectrum appears more rapidly on Ag. The *C*. *trachomatis* SERS spectrum is dominated by the vibrational features of cell surface proteins. While features attributable to specific residues and the amide backbone characterize the *C*. *trachomatis* spectrum on Ag, the corresponding SERS spectrum on Au substrates displays vibrational characteristics of aggregated proteins. The prospects for the development of a SERS based platform for rapid (<one hour), low-cost bacterial STD diagnostics are promising based on these initial studies. Furthermore, this biomedical application demonstrates the potential for SERS to be a sensitive real time probe of the dynamics of biochemical activity in the cell wall and extracellular regions of microorganisms.

## Introduction

Sexually transmitted diseases (STDs) continue to be a significant cause of morbidity in the US with ~$15.9 billion spent annually on healthcare costs related to their diagnosis and treatment^1^. Chlamydia, the most common bacterial sexually transmitted disease (STD) in the US, is caused by infection from the Gram-negative bacterium *Chlamydia trachomatis*^2^. More than 1.5 million cases of chlamydia were reported to the CDC in 2015, an increase of nearly 6% over 2014^2^. Chlamydia infection can lead to pelvic inflammatory disease, ectopic pregnancies and chronic pelvic pain in women^3^. In addition, chlamydia increases the risk of HIV transmission and infection^4^, and results in conjunctivitis, pharyngitis and pneumonia in newborns via perinatal transmission^5^.

*C*. *trachomatis* is an obligate intracellular bacterium requiring an eukaryotic host cell to complete its life cycle^6^. It has a metabolically-active, noninfectious form called a reticulated body (RB) and a metabolically-inactive but infectious form called an elementary body (EB). EBs are relatively small particles (~0.3 μm diameter) with a unique rigid, disulfide cross-linked outer membrane protein complex that helps maintain the structural integrity of the EB^6^. Following invasion of eukaryotic cells, EBs are converted to RBs, the intracellular replicating form of this organism. Approximately 20 hours after infection and subsequent multiple divisions by binary fission, the RB differentiates into the EB developmental stage and the infectious EBs are subsequently released to initiate new rounds of infection^7^.

Gonorrhea, the second most commonly reported bacterial STD in the US, results from infection by the Gram-negative bacterium *Neisseria gonorrhoeae*. Since 2011, the prevalence of gonorrhea in the US and Europe has shown a steady increase^2,8^. Patients with gonorrhea are often asymptomatic until complications arise such as pelvic inflammatory disease, ectopic pregnancy and infertility. Untreated gonorrhea can lead to disseminated gonococcal infection (DGI) when *N*. *gonorrhoeae* spreads to the blood or other parts of the body^9^. Gonococcal infections also can facilitate the transmission of HIV^10^. Since ~30% of patients infected with *N*. *gonorrhoeae* are co-infected with *C*. *trachomatis*^11^, patients treated for gonococcal infection are often routinely treated with an antibiotic regimen that is effective against *C*. *trachomatis* infection as well^12^.

Although sensitive and specific, the traditional cell culture method for chlamydia diagnosis is a technically demanding, very slow (≥72 hours) procedure rendering it unrealistic for routine and point-of-care diagnostics^13^. Similarly, *N*. *gonorrhoeae* is also a fastidious organism requiring enriched media in a CO_2_ atmosphere for lab cultured growth for ≥48 hours. Non-culture methods for STD diagnostics, such as enzyme immunoassay (EIA) and direct fluorescent antibody stain (DFA) based techniques have been recently developed^13–15^. However, nucleic acid amplification tests (NAAT) are the current best technology for chlamydia and gonorrhea diagnostics^13^. It is a growth-free diagnostic offering sensitivity and specificity comparable to culturing methods but with a faster turnaround time (~hours)^16^. However, cross-contamination, cost, inability to distinguish bacterial viability (i.e. live vs. dead cells), the presence of inhibitory factors and the need for experienced technicians in laboratory settings are NAAT limitations^17,18^. Given the asymptomatic nature of many chlamydia and gonorrhea infections, screening is recognized as the most effective approach for reducing the societal and personal impact of these diseases^13,16^. Thus, the development of alternative, low-cost, easy-to-use, point-of-care approaches for the detection and simultaneously differentiation of *C*. *trachomatis* and *N*. *gonorrhoeae* infections in clinical settings in a useful timeframe (≤one hour) for narrow spectrum antibiotic drug prescription remains a critical strategy for improving reproductive and sexual health worldwide.

Surface enhanced Raman spectroscopy (SERS) is an attractive methodology for biomedical diagnostics owing to its rapid, sensitive, specific, easy-to-use, label-free and multiplexing capabilities for molecular detection and identification^19–21^. Although SERS spectra of lab cultured bacterial cells have been reported over the past fifteen years^22–30^, there have been no previous attempts to employ this optical approach for STD bacterial pathogen diagnostics. Previous SERS efforts in our lab have resulted in the development of a nanoparticle covered SiO_2_ substrate and multivariate data analysis procedures for the acquisition and identification of 785 nm excited SERS spectra of vegetative bacterial cells^24,31–34^. Furthermore, we have identified the molecular and biochemical origins of these bacterial signals, and demonstrated their use for the diagnosis of blood and urinary tract infections^35–39^.

Here, we report on the ability of SERS to provide rapid, growth-free, detection and identification of the Chlamydia and gonorrhea etiological agents, *C*. *trachomatis* (EBs) and *N*. *gonorrhoeae*. The prospects for the development of a SERS based platform for rapid (<one hour), low-cost STD diagnostic are promising based on these initial studies. Furthermore, this biomedical diagnostic application demonstrates the potential for SERS to be a sensitive real time probe of the dynamics of biochemical activity in the cell wall and extracellular regions of these pathogenic organisms.

## Results and Discussion

### *C*. *trachomatis* and *N*. *gonorrhoeae* SERS on Au and Ag substrates

**Figure 1:**
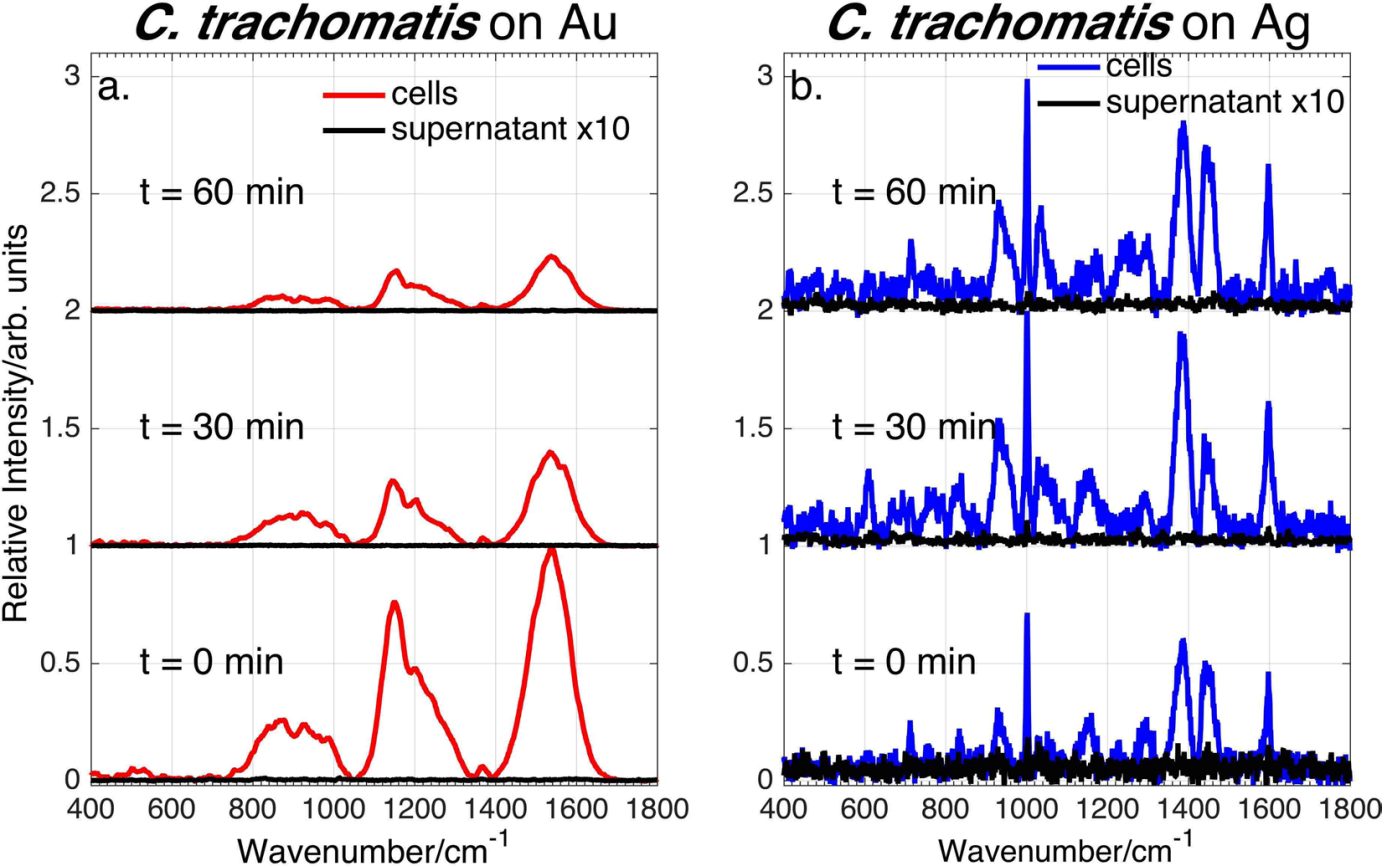
SERS spectra of *C*. *trachomatis* cells and supernatant (without cells) on Au and Ag substrates as a function of time after final (cold) water wash. Although *C*. *trachomatis* shows dramatically different SERS features on these two nanostructured metal substrates, no SERS signal is observed from the supernatant at all post washing times.

**Figure 2:**
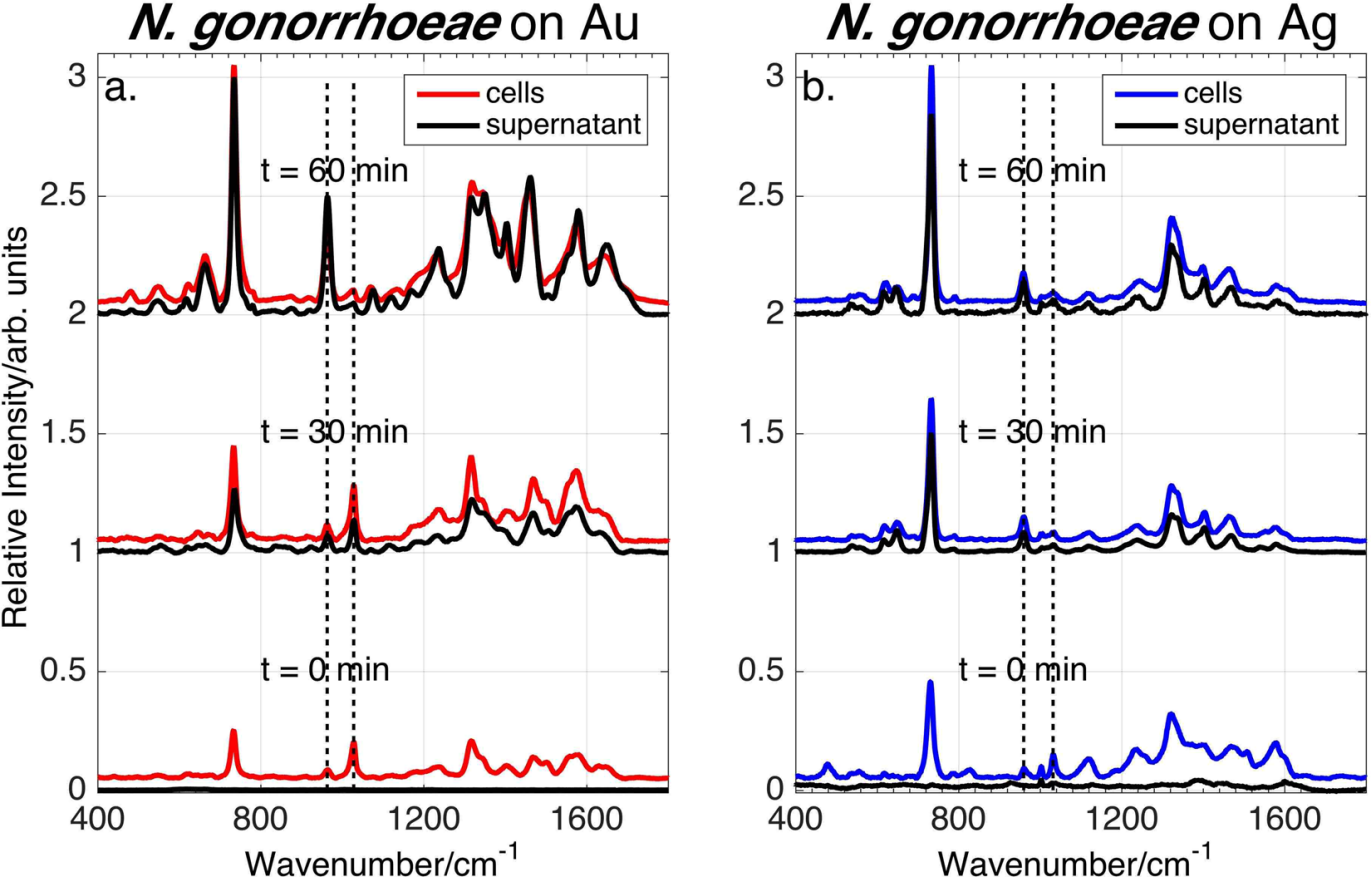
SERS spectra of *N*. *gonorrhoeae* cells and supernatant (without cells) on Au and Ag substrates. *N*. *gonorrhoeae* shows similar features on theses substrate and the supernatant signal is delayed relative to that from the cells. The dotted vertical lines highlight the disappearance of a feature at ~1030 cm^−1^ and an increase in relative prominence of a band at ~960 cm^−1^ with time.

785 nm excited SERS spectra of *C*. *trachomatis* serovar D and *N*. *gonorrhoeae* FA1090 enriched cellular suspensions on Au and Ag substrates (red and blue spectra, respectively) are shown in Figs 1 and 2, as a function of time after washing. Very different vibrational features characterize the SERS signatures of *N*. *gonorrhoeae* and *C*. *trachomatis* on both the Au and Ag substrates at all post-washing times. Thus, phenomenologically alone, these data demonstrate the potential capacity for SERS to readily distinguish these two Gram-negative STD bacterial pathogens on either Au or Ag nanoparticle substrates. Importantly, these differences are observed immediately following sample preparation (t = 0 min).

Secondly, although the SERS spectra of *N*. *gonorrhoeae* appear fairly similar on both Au and Ag substrates (Fig. 2a,b), the SERS spectra of *C*. *trachomatis* cells on Au and Ag are dramatically different. The *C*. *trachomatis* SERS spectrum (Fig. 1a) on Au exhibits three broad (~150–200 cm^−1^) spectral features centered at ~900 cm^−1^, ~1200 cm^−1^ and ~1550 cm^−1^. These unusually broad Raman features are not observed in the *C*. *trachomatis* SERS spectrum on Ag (Fig. 1b), where instead only discreet, more typically narrow (~10 cm^−1^) vibrational bands are evident with prominent bands at 936, 1002, 1035, 1385, 1446 and 1600 cm^−1^. In addition to this dramatic spectral signature difference, the relative intensity of the *C*. *trachomatis* spectrum on Ag is more than an order of magnitude weaker (counts/cell) than on Au.

As illustrated in Figs 1 and 2, the *N*. *gonorrhoeae* and *C*. *trachomatis* spectra display different *reproducible* time-dependencies on both metal substrates. The *C*. *trachomatis* signal intensity on Au decreases by ~80% in ~60 min but, in contrast, remains essentially constant on Ag over that time. The *N*. *gonorrhoeae* cell spectrum on both Au and Ag appears promptly (at t = 0 min) and increases by a factor of ~3–5 over a one hour period. However, some *N*. *gonorrhoeae* SERS vibrational frequencies and relative intensities exhibit a time dependence. For example, a band at ~ 1030 cm^−1^ evident in the early time spectra of *N*. *gonorrhoeae* on Au and Ag virtually vanishes and a ~960 cm^−1^ feature correspondingly grows in with time (dashed lines in Fig. 2a,b). Furthermore, over the course of an hour, the most intense *N*. *gonorrhoeae* vibrational band blue shifts from 731.0 cm^−1^ to 733.5 cm^−1^ on Ag and from 733.0 to 734.5 on Au (Supplementary Information Figure S1). As discussed below, these changes are attributable to biochemical activity and are spectral features that may also be used as characteristics for STD pathogen identification.

The *C*. *trachomatis* and *N*. *gonorrhoeae* SERS spectra shown in Figs 1 and 2 were obtained for sample concentrations of 10^5^ ifu/mL and 10^6^ cfu/mL, respectively, as determined by serial dilution and overnight cell culturing. Using the described sample handling protocols, the lowest *C*. *trachomatis* EB concentration to yield SERS signals were ~10^4^ ifu/mL on Au, and ~10^2^ ifu/mL on Ag substrates. The lowest concentration of *N*. *gonorrhoeae* to yield a SERS signal on the Au and Ag substrates via this simple enrichment procedure is ~10^5^ cfu/mL. (See Supplementary Information Figures S2 and S3).

### SERS spectra of *C*. *trachomatis* and *N*. *gonorrhoeae* supernatant

The SERS spectra of the (cell-free) supernatant surrounding the *N*. *gonorrhoeae* and *C*. *trachomatis* cells in the water washed suspension on Au and Ag substrates as a function of post washing time are compared to the corresponding cell suspension spectra in Figs 1 and 2. Despite the very different cell spectra on Au and Ag substrates, no SERS *C*. *trachomatis* signal can be detected from the supernatant at all post-washing times on both metal surfaces (Fig. 1). The requirement that molecules be ≤ ~5 nm from nanostructured substrates for effective plasmonic enhancement eliminates molecules in the *C*. *trachomatis* cytoplasm as being the source of the observed cellular SERS signatures. Thus, the SERS spectra of *C*. *trachomatis* are only attributable to cell wall features.

In contrast, the SERS spectra of the *N*. *gonorrhoeae* supernatant on both Au and Ag (Fig. 2) exhibit a time-dependent signature that becomes nearly equivalent to the cell SERS spectrum after ~30–60 min. Following the final water washing (t = 0 min), no significant SERS spectrum is observed from the *N*. *gonorrhoeae* supernatant while the *N*. *gonorrhoeae* cells exhibit a strong signal on both Au and Ag substrates (Fig. 2a,b). Thus, this early time spectrum originates from *N*. *gonorrhoeae* cell wall or cell wall associated molecules. At longer times (30 and 60 min), supernatant and cell SERS spectra appear to be nearly identical. Thus, unlike *C*. *trachomatis*, the *N*. *gonorrhoeae* SERS spectra at these later times result from molecules that have been secreted into the supernatant.

**Figure 3:**
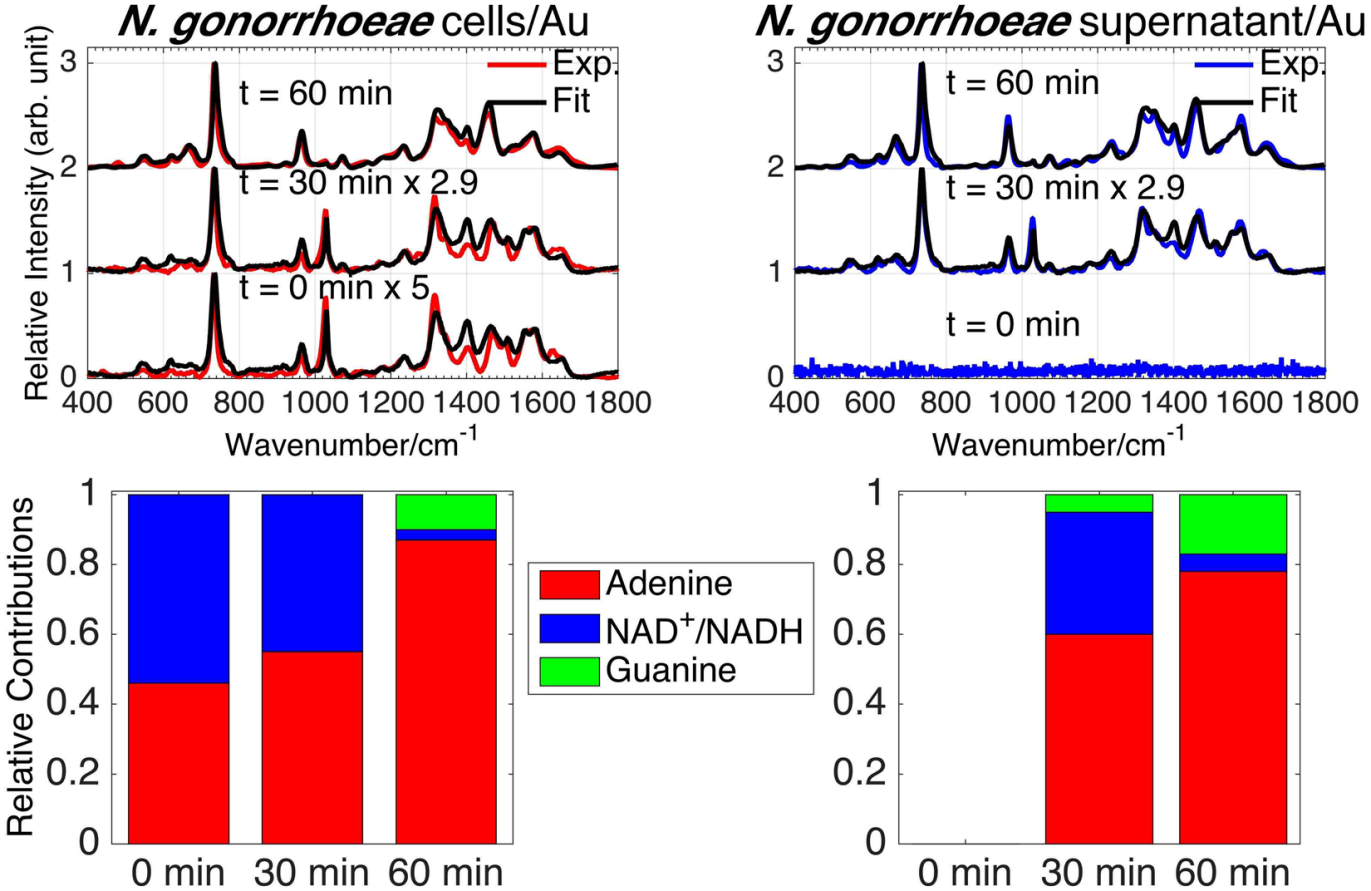
Observed and best-fit SERS spectra of *N*. *gonorrhoeae* cells and supernatant as a function of post washing time on Au substrates (top left and right, respectively). Bar graphs show the corresponding best-fit determined relative amounts of adenine, NAD^+^/NADH and guanine (normalized spectra) at each post washing time for the *N*. *gonorrhoeae* cells and supernatant spectra.

**Figure 4:**
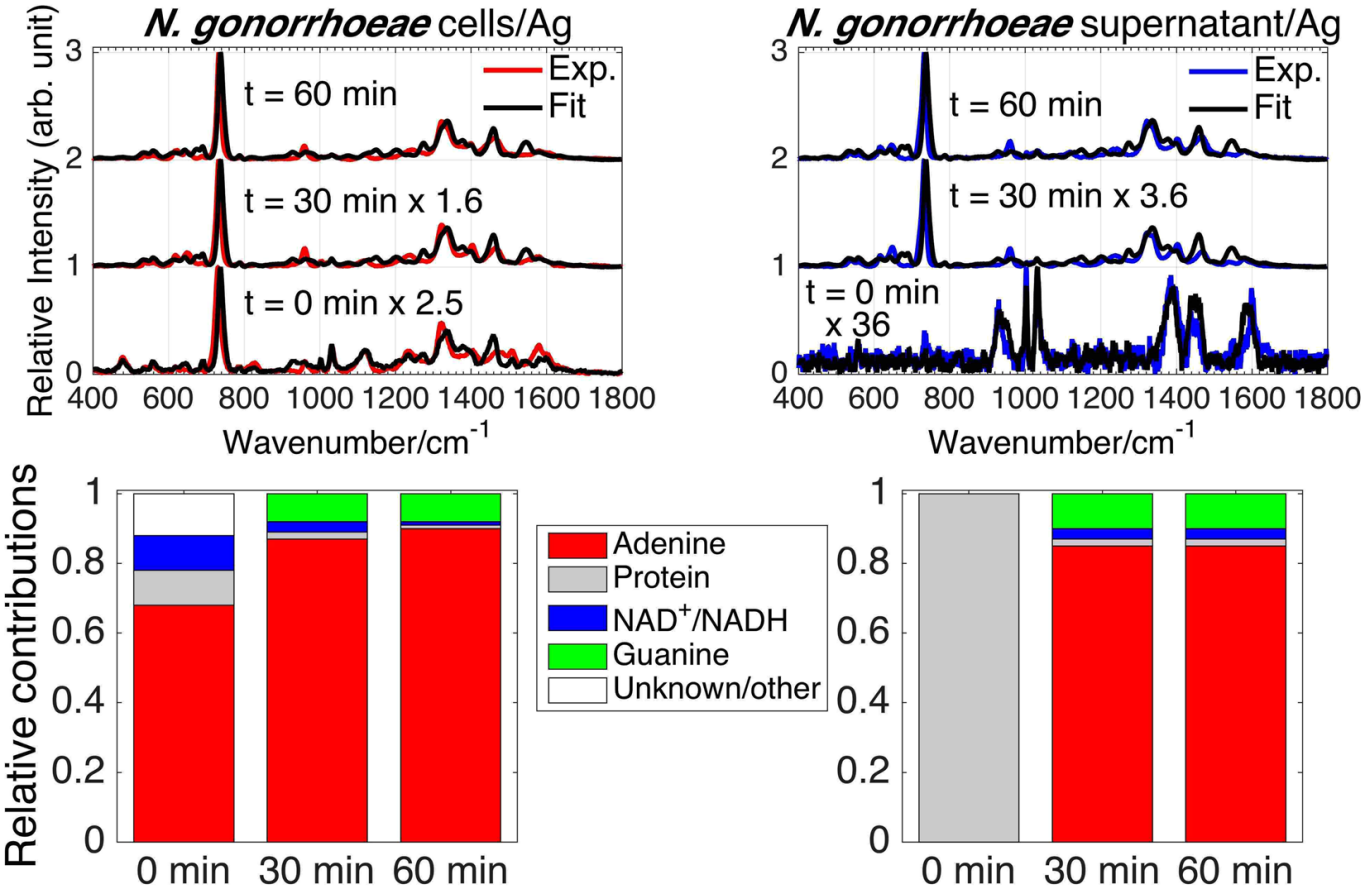
Observed and best-fit SERS spectra of *N*. *gonorrhoeae* cells and supernatant as a function of post washing time on Ag substrates (top left and right, respectively). Bar graphs show the corresponding best-fit determined relative amounts of adenine, NAD^+^/NADH and guanine, protein and some unknown components (normalized spectra) at each post washing time for the *N*. *gonorrhoeae* cells and supernatant spectra.

While the results shown in Figs 1–4 are for averaged spectra (~10) of a single bacterial growth, we have obtained virtually identical results at each of the SERS acquisition times for all of the independent bacterial growths we have obtained. The standard deviation of normalized averaged SERS spectra of multiple growths of *N*. *gonorrhoeae* (four/three growths for cell spectra on Au/Ag; two/three growths for supernatant spectra on Au/Ag) are shown in Figure. S4. The *N*. *gonorrhoeae* SERS spectra and their time dependence are highly reproducible and the results described here are thus not growth dependent. SERS spectra of four independent growths of *C*. *trachomatis* also show a high degree of reproducibility (see below).

### Molecular origins of *N*. *gonorrhoeae* SERS spectra

**Figure 5:**
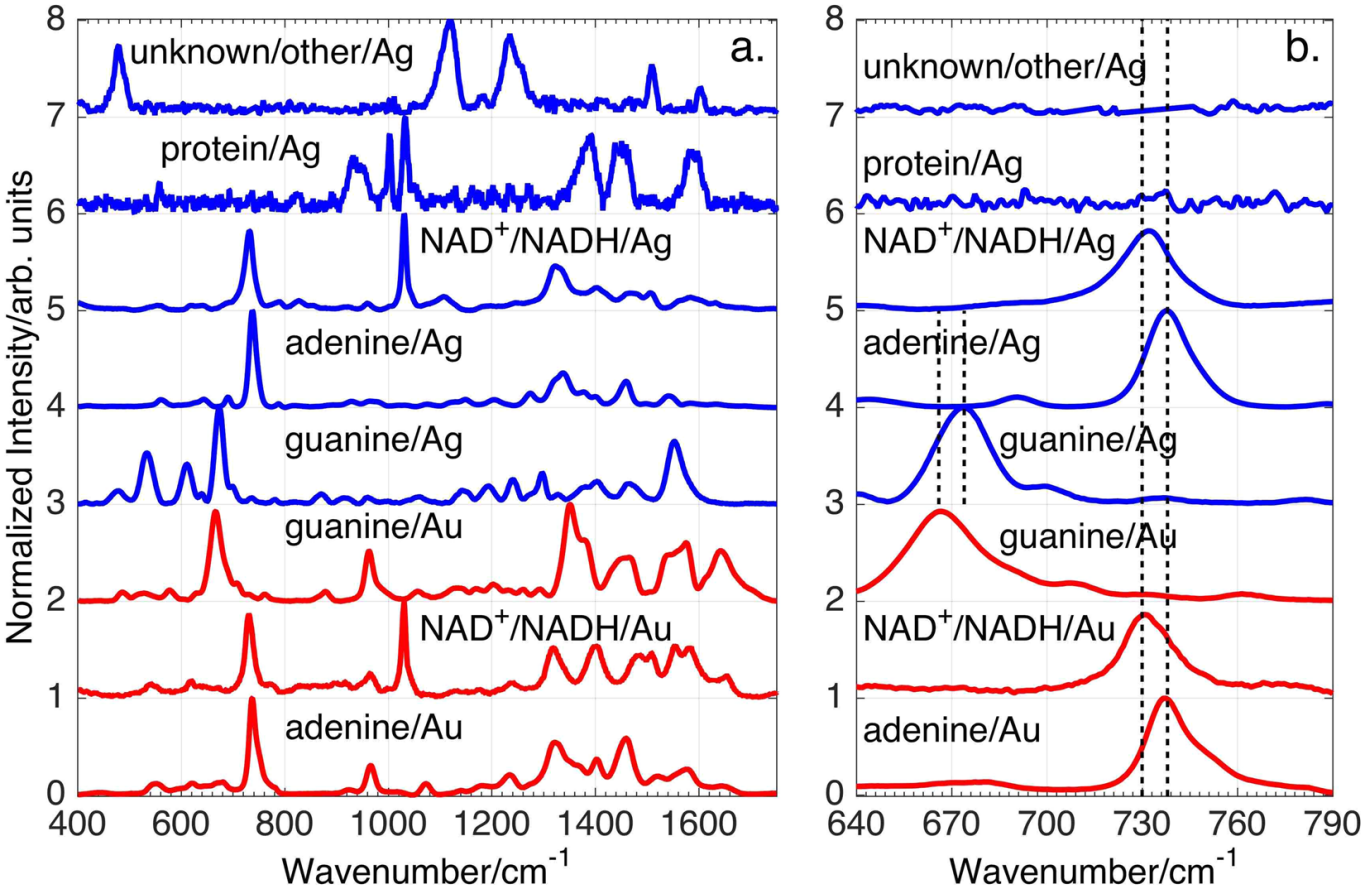
(**a**) Normalized SERS spectra of the molecular components identified in the SERS spectra of *N*. *gonorrhoeae* on Au and Ag: nicotiamide adenine dinucleotide (NAD^+^/NADH), adenine, and guanine. (**b**) An expanded view of the SERS spectra of these compounds in the 640–790 cm^−1^ range where characteristic small frequency shifts of the strongest bands are evident.

As previously shown, the 785 nm excited SERS spectra of bacteria are dominated by the contributions of purines or purine containing metabolites, such as adenine, hypoxanthine, guanine, xanthine, uric acid, guanosine, and AMP in the exogenous cellular region resulting from the metabolic degradation of nucleotides and nuclei acids as part of the rapid bacterial starvation response^38,39^. In these prior studies a spectral best-fitting procedure was used to determine the relative contribution of these purines to the observed bacterial SERS spectra. The same^38,39^ best-fitting procedure is employed here to identify the key molecular species that account for the *N*. *gonorrhoeae* SERS spectra as a function of time (Fig. 2). The resulting best fits to the observed *N*. *gonorrhoeae* SERS spectra of cell and supernatant (cell-free) samples on Ag and Au substrates at 0, 30 and 60 min post washing are displayed in Figs 3 and 4, respectively. Excellent fits based on Raman frequencies and relative intensities are found by including contributions primarily from adenine, guanine, and nicotinamide adenine dinucleotide (NAD) for the *N*. *gonorrhoeae* SERS spectra on Au substrates. On Ag substrates, a protein contribution (*vide infra*) is detected, in addition to these same three dominant purine components. Some unidentified components are evident at the earliest time on Ag. The normalized SERS spectra of the molecular components used for this fitting procedure are shown in Fig. 5. A summary of the molecular origins of the purinergic vibrational bands observed in the *N*. *gonorrhoeae* SERS spectra is given in Table 1 based on these individual molecular component spectra (Fig. 5). Characteristic vibrational bands, for example, adenine’s 734 cm^−1^ ring stretching mode, guanine’s 664 cm^−1^ ring stretching mode or the 1030 cm^−1^ nicotinamide ring breathing mode of NAD^+ ^^40^, distinguish most of these molecular contributors to the *N*. *gonorrhoeae* SERS spectra. The SERS spectra of the oxidized NAD^+^ and reduced NADH forms of this co-enzyme are identical on our substrates (Supplementary Figure S5). Both exhibit the NAD^+^ characteristic ~1030 cm^−1^ band^40^. However, since this vibrational frequency is absent in known NADH vibrational spectra^40^ either only NAD^+^ contributes to these the *N*. *gonorrhoeae* SERS spectra or the incompletely reduced Au^+3^ and Ag^+^ metal ions efficiently oxidizes all NADH. Thus we denote this component as NAD^+^/NADH (Figs 3–5). Normalized SERS spectra of a typical protein, human serum albumin (HSA) and the residual unknown component are also shown in this figure. Finally, it should be noted that small but reproducible frequency shifts and some relative intensity changes (e.g. absence of 960 cm^−1^ band in adenine on Ag spectrum) are metal dependent (Fig. 5).

**Table 1:** Vibrational assignments for *N*. *gonorrhoeae* SERS spectra.

Peak Position Au/Ag (cm^−1^)	Molecular Assignment
481/481	Guanine
548/548	adenine/NAD^+^
622/621	Adenine
664/672	Guanine
733/731	NAD^+^
735/734	adenine
962/960	adenine/guanine/NAD^+^
1028/1032	NAD^+^
1236/1240	adenine
1316/1322	adenine/NAD^+^
1350/1339	guanine
1404/1401	adenine
1460/1469	adenine
1580/1580	adenine/guanine
1646/1606	guanine

The best-fit determined relative contributions of the normalized SERS molecular components to the observed SERS spectra of the *N*. *gonorrhoeae* cellular suspensions and the supernatant on both Au and Ag substrates as a function of time are summarized by the bar graphs in Figs 3 and 4. Some consistent trends and striking differences are observed in this time dependent SERS analysis. Initially (t = 0 min), no signal is detected on Au and only a very weak protein signature is observed on Ag in the supernantant only samples. By 60 min, both the cellular suspension and supernatant SERS spectra are nearly identical in terms of relative contributions of adenine, guanine and NAD^+^/NADH with adenine making the largest contribution at least in terms of this normalized analysis. Some observed systematic trends are the relative disappearance of NAD^+^ features and the growth of adenine and guanine signals with time (see Figures S1, 2–4). The loss of the ~1030 cm^−1^ band intensity, the small blue shift in the most intense feature from 731/733 to 734/735 cm^−1^ (Au/Ag) and the growth of the 960 cm^−1^ and ~664 cm^−1^ bands are indicators of these time-dependent purinergic effects. Finally comparison of Figs 3 and 4 reveal that the *N*. *gonorrhoeae* SERS spectra appear to evolve with a slower time dependence on Au substrates than on Ag surfaces. This appears to be a general characteristic of bacterial SERS spectra on the substrates used for these studies and may be attributable to the different perturbing effects of these metals or the different rates of metal-purine association in the exogenous metabolome of these cells. This will be the subject of a subsequent report. The relative contributions of normalized components to the *N*. *gonorrhoeae* SERS spectra (bar graphs in Figs 3 and 4) can be converted to relative number densities via the relative SERS susceptibilities given in Supplementary Figure S6 assuming no complexing or special solvation effects in the region of the bacterial cells.

**Figure 6:**
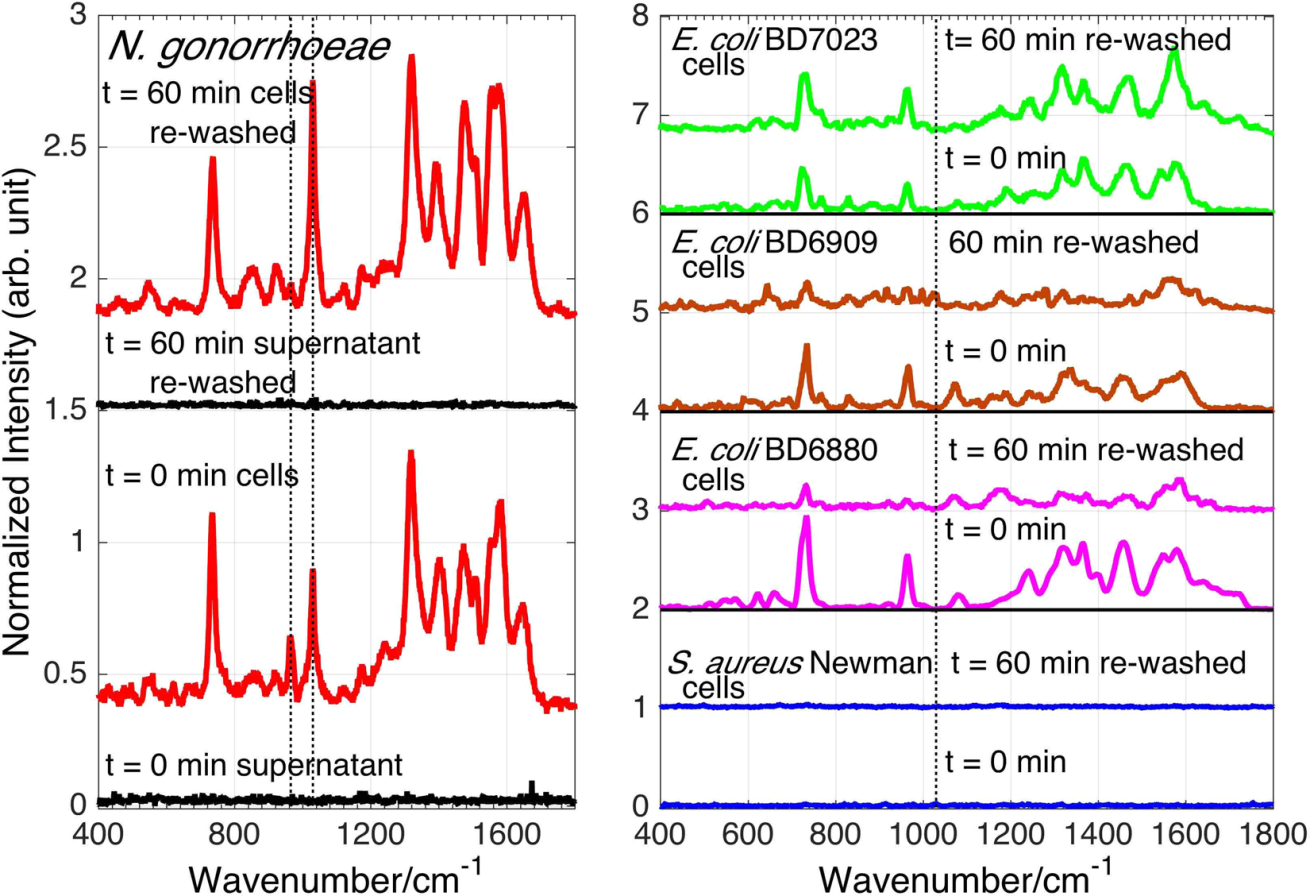
SERS spectra of *N*. *gonorrhoeae* cells and supernatant on Au substrates at t = 0, immediately after enrichment are compared with the same sample after 60 min and rewashing in water (LHS). Dotted vertical lines at 960 cm^−1^ and 1030 cm^−1^ correspond to adenine and NAD^+^/NADH contributions. The re-washed 60 min spectrum only reveals NAD^+^/NADH associated with the cells. SERS spectra of cellular suspensions of 4 different bacterial species, 1 *S*. *aureus* and 3 *E*. *coli* strains, are correspondingly compared at t = 0 and for rewashed 60 min preparations, and no NAD^+^/NADH can be detected in any of these spectra (dotted vertical black line) (RHS).

Interestingly, despite its unequivocal presence in these *N*. *gonorrhoeae* SERS spectra, NAD^+^/NADH had not been identified in previously analyzed 785 nm excited bacterial SERS spectra^38,39^. To further probe the origin of this SERS contribution and assess its value for *N*. *gonorrhoeae* diagnostics, SERS spectra of an *N*. *gonorrhoeae* sample which had been left at room temperature for 60 min and re-washed with ice-cold water were obtained. The supernatant and cellular suspension SERS spectra of this 60 min rewashed sample on Au substrates are compared in Fig. 6 to the initial (t = 0 min) spectra (Fig. 3). The 60 min rewashed *N*. *gonorrhoeae* supernatant on Au exhibits no SERS spectrum as found for the original t = 0 min sample (Fig. 3). All purine metabolites in the exogenous region after 60 min of the initial water washing procedure have been removed by this procedure. Furthermore, in contrast to the 60 min spectrum of the original cellular sample attributable to adenine, guanine and NAD^+^/NADH (Fig. 3), the 60 min rewashed *N*. *gonorrhoeae* cellular suspension SERS spectrum is appears to be NAD^+^/NADH only with little detectable adenine and guanine. The increased relative intensity of the characteristic NAD^+^ 1030 cm^−1^ band and the decreased relative intensity of the adenine 960 cm^−1^ feature (dashed vertical lines Fig. 6), are indicative of this predominant NAD^+^ character of these re-washed cellular suspension spectra. (Supplementary Figure S7). Thus, these results are consistent with NAD^+^/NADH being located in the outer bacterial cell membrane region.

Furthermore, on the right hand side of Fig. 6, SERS spectra of the 60 min rewashed cell suspensions of some *E*. *coli* strains (Gram negative) and an *Staphylococcus aureus* strain (Gram positive) are compared with their corresponding t = 0 SERS results and with the *N*. *gonorrhoeae spectra* on Au substrates. These other bacterial organisms were also grown in the same supplemented culture media. As seen in Fig. 6 (RHS), the absence of the 1030 cm^−1^ band in these spectra (black vertical line) indicates that the appearance of the cell outer layer associated NAD^+^/NADH in *N*. *gonorrhoeae* SERS spectra is further evidence that this SERS molecular component is not due to a sample preparation artifact such as residual growth culture media impurity and, at least in this limited comparison, is a unique property of *N*. *gonorrhoeae*. Since this NAD^+^ signature has not been evident in any other bacterial SERS spectrum that we have observed with 785 nm excitation^38,39,41^, the presence of this membrane bound or associated marker appears crucial for the unique identification of this pathogen and hence to the value of a SERS based platform for bacterial STD diagnostics. NAD^+^/NADH is co-substrate for the known *N*. *gonorrhoeae* membrane-bound enzymes, NADH dehydrogenase and NAD(P)H oxidase, that are critical for the growth of this organism in the human body and thus may account for the observed bounded NAD/NADH SERS signals for this species^42,43^.

### Molecular origins of *C*. *trachomatis* SERS spectra

As discussed above, the molecules accounting for the *C*. *trachomatis* SERS spectra are securely located on the outer wall region of the EBs and are not in the exogenous supernatant in contrast to the origins of the *N*. *gonorrhoeae* SERS signatures (Fig. 2) and exhibit completely different, seemingly uncorrelated, characteristic spectral signatures on Au and Ag (Fig. 1). Unlike other Gram-negative bacteria, such as *N*. *gonorrhoeae*, the *C*. *trachomatis* EB cell envelope has two unusual features^44^. First, the typical peptidoglycan layer between the outer and an inner cell membrane is replaced by a supramolecular structure of cysteine rich proteins. Secondly, *C*. *trachomatis* EBs have unique outer membrane surface protein complexes characterized by highly cross-linked disulphides that are thought to convey structural integrity particularly against intracellular osmotic stress^45–47^. As additional evidence implicating cell surface proteins as the origin of the *C*. *trachomatis* SERS spectrum, the observed discrete features for the Ag substrate spectra (see Table 2) match previously reported characteristic protein SERS vibrational frequencies^48–52^. These band assignments correspond to aromatic residues, particularly phenylalanine, and peptide backbone motions.

**Table 2:** Vibrational Assignment for *C*. *trachomatis* SERS spectra on Ag.

Peak Position (cm^−1^)	Proposed Assignment^*^
610	ν(CS)
756	Trp
931	C-C, C-COO^−^
1002	Phe ring breathing (ν_12_)
1035	Phe in-plane ring CH def.
1386	COO^−^
1448	CH_2_ deformation
1599	Phe ring stretching

*Band assignments from refs^49–53^.

**Figure 7:**
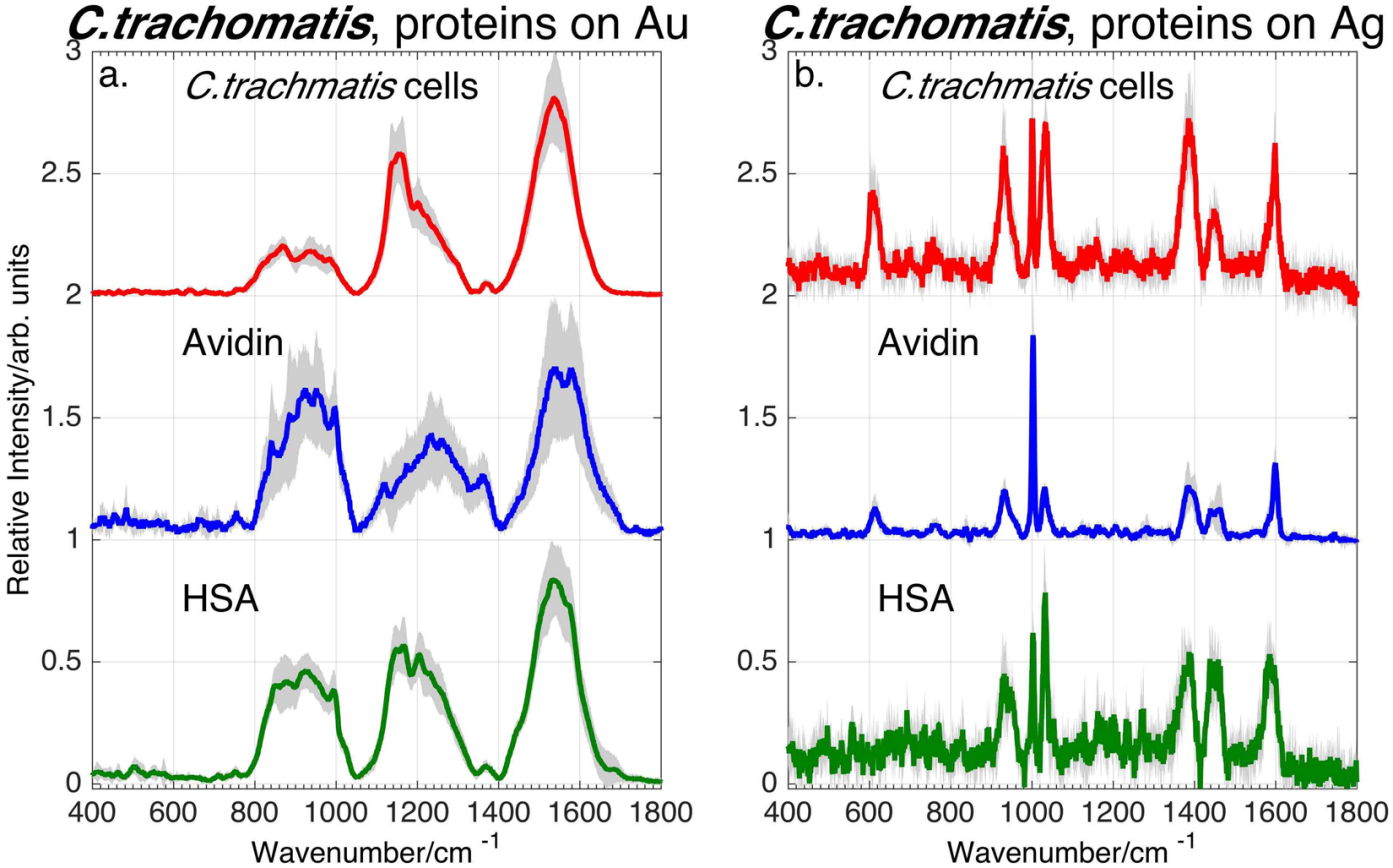
SERS spectra of *C*. *trachomatis* cells (from four growths), and aqueous solutions of the proteins human serum albumin (HSA) and avidin on (**a**) Au and (**b**) Ag substrates. The shaded areas indicate the standard deviation at each frequency to demonstrate the reproducibility of these spectra.

As a test of this hypothesis, SERS spectra of several proteins on Au and Ag substrates were obtained and compared to *C*. *trachomatis* spectra. Solutions of these proteins were prepared following the same preparation protocols used for the *C*. *trachomatis* samples. SERS spectra of two of these proteins in aqueous solution on Au (100 µM) and Ag (10 nM) substrates, human serum albumin (HSA) and avidin, are compared to those of *C*. *trachomatis* from four growths in Fig. 7. The grey shading corresponds to ± one standard deviation of ~ten spectra of HSA and avidin, and from the *C*. *trachomatis* multiple growths relative to the averaged spectra (solid lines) and demonstrate the robustness of these SERS signatures. The different concentrations indicate the different relative SERS susceptibilities for proteins on the two metals as noted above for the *C*. *trachomatis* spectra. HSA and avidin are similarly sized proteins (~66–67 kDa) that are dominated by high alpha-helical and beta-sheet secondary structural content respectively. Despite these structural differences, the similarity between the protein and the *C*. *trachomatis* SERS spectra, as well as the strong metal dependence is clearly evident. The same three unusually broad bands centered at ~900 cm^−1^, ~1200 cm^−1^ and ~1550 cm^−1^ characterize the SERS spectra on Au, and narrow spectral features at ~931, 1002, 1035, 1386, 1448 and 1599 cm^−1^ are seen in the Ag substrate spectra for all three samples. Consistently similar SERS spectra have been observed for all other proteins and polypeptides we have obtained. (See Supplementary Figure S7). Thus, these results indicate that, in contrast to *N*. *gonorrhoeae*, the SERS spectra of *C*. *trachomatis* arise from protein molecules on or associated with the *C*. *trachomatis* cell surface.

The unusual breadth and robust center frequency of the protein and *C*. *trachomatis* vibrational bands on Au substrates (Figs 1 and 7) suggest that these features may be due to protein aggregation. Dynamic light scattering (DLS) measurements were carried out to determine protein size distributions in these solutions and then correlated with observed SERS spectra. This protein analysis is discussed in greater detail in a subsequent report. However, we note here that both the % mass of particles with larger radii and the observed broad SERS intensity of HSA and avidin on Au increased after vigorous mechanical agitation which is known to enhance protein aggregation^53^. (Supplementary Figure S8) Minimal and vigorous pipetting was used to generate the range of both shearing stress and cavitation resulting in induced aggregation. Thus, the correlated increase in particle sizes and broad SERS spectral intensity on Au strongly indicates that these anomalously broad SERS spectra of proteins, and by extension *C*. *trachomatis*, are due to protein aggregates created during the solution preparation process. During the *C*. *trachomatis* enrichment protocol, this must occur when the centrifuged bacterial cells are re-suspended in water after each centrifugation step and pipetting is used to disperse the cell pellet apparently resulting in the aggregation of the proteins on the outer layer of the cell membrane. The observed decrease of the *C*. *trachomatis* SERS intensity as a function of post washing time (Fig. 1) may arise from the structural relaxation of the agitation-induced protein aggregates on the cell surface.

In contrast, only narrower vibrational features with more typical vibrational widths and characteristic protein vibrational are found in the protein and *C*. *trachcomatis* SERS spectra on Ag. The assignments for the observed *C*. *trachcomatis* SERS bands on the Ag substrate are given in Table 2. These features correspond to characteristic vibrational transitions in aromatic amino acid residues (phenylalanine, tryptophan) and other characteristic polypeptide molecular moieties (C-S stretch, and CO_2_^−^ and CH_2_ bending motions). The absence of this protein cluster features in the SERS spectra of *C*. *trachcomatis* and proteins is attributed to the more strongly perturbative interaction of Ag nanoparticles and residual Ag^+^ ions with macromolecular structures and proteins more specifically^54–56^.

Despite the qualitative similarity between the *C*. *trachcomatis* and protein SERS spectra (Fig. 7), these spectral signatures can still be used to identify this STD pathogen on both metals. For example, the consistent differences in the relative SERS peak intensities for resulted in a cross-validated partial least squares discriminant analysis (PLS-DA) treatment^39^ of the HSA, avidin and *C*. *trachcomatis* SERS spectra successfully classified these samples with 99/97% sensitivity and 98/96% specificity for Au/Ag substrates. (See Supplementary Figure S9) Furthermore, from a diagnostics perspective, protein and *C*. *trachcomatis* would be separated by the centrifugation preparation step, at least for samples prepared by this current protocol.

### Biochemical distinctions evident in SERS of STD pathogens

The distinct SERS spectral signatures of these two Gram-negative bacterial STD pathogens reflect their very different “lifestyles”. All previously analyzed and observed bacterial SERS spectra are dominated by the purine metabolites of nucleotide degradation (adenine, guanine, hypoxanthine, xanthine, guanosine, uric acid, AMP) in the exogenous region of cells resulting from the rapid starvation response of bacterial cells upon being placed in pure water washes following enrichment from nutrient rich environments^38,39^. The absence or presence of different degradation/salvage enzymes in the known purine metabolism pathways of these organisms plays a central role in determining the bacterial specificity of these purine-based SERS signatures along with strain/species specific enzyme turnover rates, secretion kinetics, and catalytic feedback mechanisms^38,39^. Examination of the purine metabolic pathways for *N*. *gonorrhoeae*^57^ (see Figure S11) shows that enzymes required for the degradation of nucleotides resulting in adenine and guanine, but not hypoxanthine, uric acid, or xanthine, are present in this bacterial strain, consistent with the SERS spectral fitting analysis (Fig. 4). This result is also consistent with purine metabolic pathway dependence we have described previously for more than 20 other bacterial species and strains^38,39^. The time dependent release/appearance of these degradation products (adenine and guanine) into the extracellular region accounts for the time dependent *N*. *gonorrhoeae* SERS spectral features reported here (Figs 2–4, S1). Most notably the relative decrease of ~1030 cm^−1^ band intensity, the 731/733 cm^−1^ to 734/735 cm^−1^ (Au/Ag) blue shifts and the growth of the 960 cm^−1^ and ~664 cm^−1^ bands are all consistent with the slower appearance of the free base extracellular metabolites adenine and guanine relative to NAD^+^/NADH, which is shown here to be more strongly associated with the cell membrane (Fig. 6). It should be noted the NAD^+^ signature distinguishes the *N*. *gonorrhoeae* SERS spectra from all other bacterial SERS spectra we have thus far analyzed for molecular components^38,39^.

*C*. *trachomatis* is the only bacterial species we have studied that has not exhibited any purinergic components in its SERS spectrum (or a supernatant SERS spectrum). Unlike the previously studied bacterial species, *C*. *trachomatis* is an obligate intracellular pathogen that depends on the host cell for nutrient and energy supply as noted above and its purine metabolic pathway reflects its parasitic lifestyle. *C*. *trachomatis* lacks all enzymes for the *de novo* synthesis of AMP, the key molecule to the purine degradation pathway and is missing the genes coding for phosphoribosyl transferases, the key group of enzymes responsible for escorting degraded free bases into the exogenous cell region^7,57^. In other words, isolated *C*. *trachomatis* cells are unable to produce the purine metabolites via nucleotide degradation pathways and therefore, in contrast to *N*. *gonorrhoeae* and all the other bacteria we have studied via SERS, no purine metabolites contribute to its SERS spectra. The purine nucleotide metabolic pathways of these two gram negative bacteria are contrasted in Figure S11. In the absence of any of these free base purine metabolites in the pericellular region, the SERS spectrum of *C*. *trachomatis* is then dominated by the protein signature of the cell wall envelope.

## Conclusion

SERS spectra of *C*. *trachomatis* and *N*. *gonorrhoeae*, the two most common bacterial STD pathogens, have been obtained for the first time and demonstrate the potential of a SERS-based platform for rapid, label and growth-free detection and identification of these causative agents. The unique SERS vibrational signatures on Au and Ag substrates distinguish these two bacteria and is the basis for this identification methodology. The ultimate time scale for a rapid SERS-based diagnostic platform will probably be dependent on the speed of the sample enrichment protocol, however, when combined with a portable instrument and an effective bacterial cell enrichment procedure, a species specific, growth-free STD diagnostic may potentially be achieved in less than one hour via a SERS-based platform. The *N*. *gonorrhoeae* SERS signature, both in the cellar suspension and its supernatant, is dominated by adenine, guanine and NAD^+^. The free nucleobases result from the bacterial starvation response. Thus, given the origin of these signals arising from this metabolic cellular response, live vs. dead *N*. *gonorrhoeae* cell discrimination should be possible via SERS potentially providing better real time treatment efficacy and disease prognosis than NAAT based approaches, and will be the subject of subsequent studies. In contrast, the *C*. *trachomatis* spectrum is due to proteins on the cell surface only. Interestingly, on Au substrates this cell surface vibrational signature is dominated by protein aggregates but on Ag, this aggregate spectrum is absent and only specific residue and backbone features are detected. Chlamydial loads determined by genotype-based techniques in first-void urine specimens in infected patient range from ~10^1^ to 10^5^ EB/mL, and ~10^4^ EB/mL in vulvo-vaginal swabs^58,59^. Therefore the demonstrated diagnostic sensitivity of SERS for chlamydial EB identification (10^2^–104 ifu/mL) falls within this range of EB concentrations in patient samples. *N*. *gonorrheoae* loads found in infected patient urine has been reported in the ~10^4^–106 cfu/mL^60^. This range overlaps with the SERS sensitivity demonstrated here (~10^5^ cfu/mL), and enrichment procedure improvements should readily allow higher *N*. *gonorrhoeae* diagnostic sensitivities to be achieved. Finally, aside from demonstrating here the potential of SERS methodology for a disease specific (STD) diagnostic platform, SERS also serves as a novel bioanalytical tool for studying bacterial real time metabolic and enzymatic processes in the extracellular and near outer cell wall regions.

## Methods

### SERS substrates

All SERS spectra reported here were obtained using previously developed *in-situ* grown, aggregated Au or Ag nanoparticle covered SiO_2_ substrates^24^. These substrates are produced by a metal ion doped sol-gel procedure resulting in small (2–15 particles) aggregates of monodispersed ~80–100 nm Au or Ag nanoparticles covering the outer layer of ~1 mm^2^ SiO_2_ substrate. Characterization of their performance for providing reproducible SERS spectra of bacteria have been described in prior reports^24,32,36^.

### *In vitro* cultivation of *C. trachomatis*

McCoy cell line (CRL-1696, ATCC), used as the host cells for the cultivation of *C*. *trachomatis* D/UW-3/CX (VR-885, ATCC), are grown in MEM containing 10% fetal bovine serum in a humidified incubator at 37 °C and 5% CO_2_ until 90–100% confluent. The *C*. *trachomatis* infected McCoy cells are maintained in a special medium consisting of 90 mL DMEM (Life Technologies), 10 mL fetal bovine serum and 1 μg/mL cycloheximide. Further details on *C*. *trachomatis in vitro* cultivation and harvest are described in supplementary information. Aliquots of harvested *C*. *trachomatis* were stored in Hank Balanced Salt Solution (HBSS) at −80 °C.

#### C. trachomatis sample preparation protocols

Thawed harvested *C*. *trachomatis* cells were centrifuged (23,000 *g* for 4 min at 4 °C) and the HBSS supernatant removed. The resulting pellet predominantly consisted of *C*. *trachomatis* EBs with a small amount of RBs and was subsequently washed four times with 10 µL of ice-cold deionized water. The sample is centrifuged (23,000 *g* for 4 min at 4 °C) between washes. Any remaining RB cells are lysed and removed during the washing process. After the last wash, the EB pellet is dispersed in 5 µL of ice-cold deionized water by pipetting and vortexing.

#### N. gonorrhoeae growth and preparation protocols

*N*. *gonorrhoeae* (FA1090, ATCC) are grown on chocolate II agar plates with IsoVitaleX enrichment (BD). The cultivation and harvest procedure for this bacterial strain is described in detail in the supplementary information. The bacteria are washed four times with 0.5 mL ice-cold water and centrifuged at ×4500*g* at 4 °C between each wash. After the last wash the bacterial pellet is dispersed in 50 µL of ice-cold water by vortexing. SERS spectra were acquired from multiple growths of *N*. *gonorrhoeae* on Au and Ag substrates. More specifically, three and four growths of *N*. *gonorrhoeae* cellular suspensions on Ag and Au substrates, and the supernatant of three and two *N*. *gonorrhoeae* independent growth were obtained in total on Ag and Au substrates (Figure S4).

### SERS spectral acquisition and data processing

A 1 µL sample is placed on the Au or Ag SERS substrate and air-dried for ~5 min prior to signal acquisition. All SERS spectra were acquired with an RM-2000 Renishaw Raman microscope employing a 50x objective and 785 nm excitation. Incident laser powers of ~3.3 mw and ~10 S of illumination time were used to obtain the reported bacterial SERS spectra. Typically ten spectra per sample were obtained for each experimentally reported spectrum. GRAMS/AI™ Spectroscopy Software was used to manually baseline correct the experimental SERS spectra. The illuminated Raman excitation area was ~30 μm × 6 μm. The incident fluence used for these studies is thus well below threshold for any laser tweezing effects to be evident. Peak frequency precision is ±0.5 cm^−1^.

A PLS toolbox (v7.3.1) from Eigenvector Research, Inc. (WA, USA) in a Matlab platform was used for the PLS-DA analysis resulting in Figure S9. For this analysis, the baseline corrected observed spectra were normalized to their peak intensity maxima and converted to barcodes, i.e. a series of “ones” or “zeroes” based on the sign of the second derivative at each wavenumber, to use as input vectors for this PLS-DA classification procedure. As demonstrated previously, this barcode procedure results in improved sensitivity and specificity relative to using spectra (or first or second derivatives) as input vectors for multivariate data analysis treatments^33^. Optimized PLS-DA classification performance resulted for a model which included 3/5 latent variables (LV) which corresponded to a minimum in root mean square error (RMSE). Cross-validation on the data set was accomplished by the random subset method employing 7/8 different test sets and 10/10 iterations for SERS spectra on the Au/Ag substrates.

### Data availability statement

All data generated or analysed during this study included in this published article (and its Supplementary Information files) are available from the corresponding author on reasonable request.

## Electronic supplementary material

Supplementary Information
