# Supplementary material for: Surface enhanced Raman spectroscopy of Chlamydia trachomatis and Neisseria gonorrhoeae for diagnostics, and extra-cellular metabolomics and biochemical monitoring
Source: Sci Rep. 2018 Mar 26;8:5163. doi: 10.1038/s41598-018-23562-5 (PMC5980109; doi:10.1038/s41598-018-23562-5)
Supplement: Supplementary file 1 — Supplementary Information [file 41598_2018_23562_MOESM1_ESM.pdf]

Supplementary Information

Surface enhanced Raman spectroscopy of *Chlamydia trachomatis* and *Neisseria gonorrhoeae* for diagnostics, and extra-cellular metabolomics and biochemical monitoring

Y. Chen, W. R. Premasiri, and L. D. Ziegler

Department of Chemistry and The Photonics Center, Boston University, MA, 02215

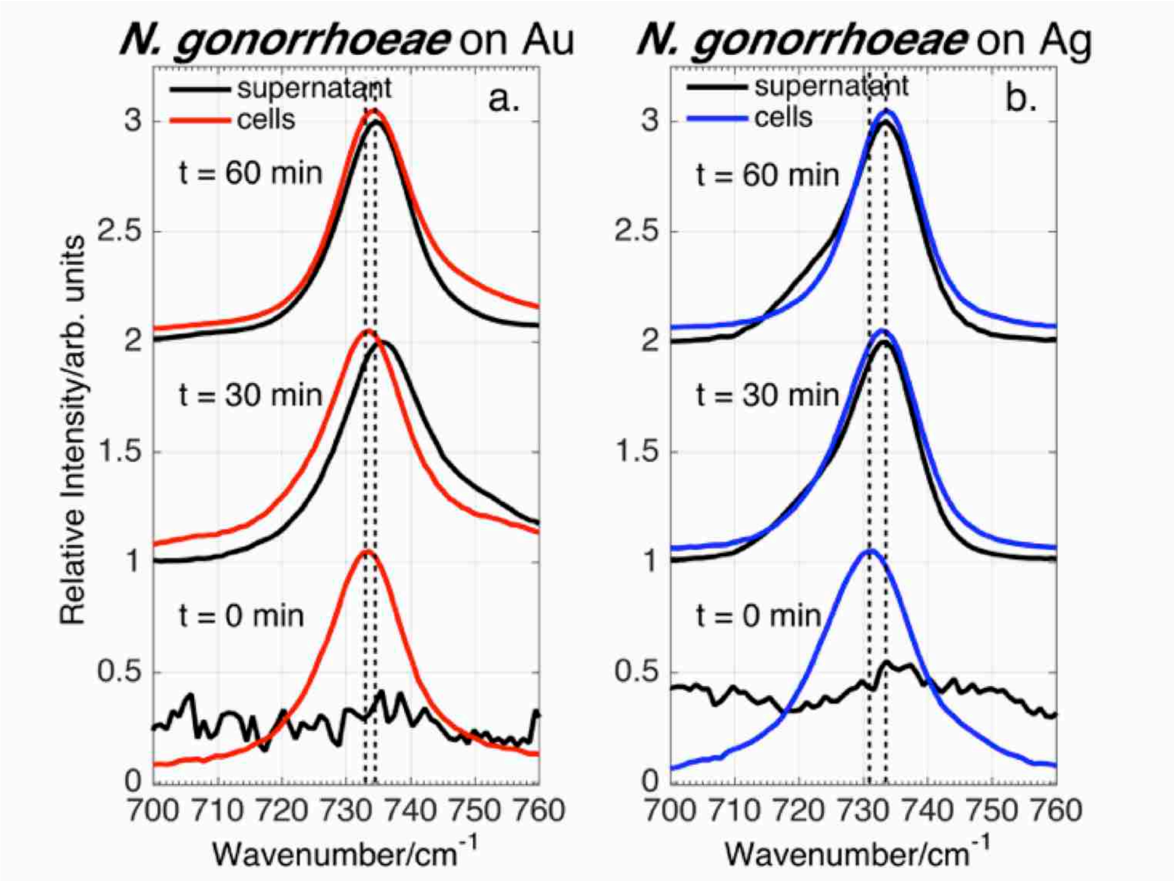

Supplementary Figure S1. The most intense *N. gonorrhoeae* vibrational band blue shifts from 731.0 cm^{-1} to 733.5 cm^{-1} on Ag and from 733.0 to 734.5 on Au as a function of time. This is due to the increase of the contribution of adenine relative to NAD^{+} to the SERS spectra of *N. gonorrhoeae* over this time period.

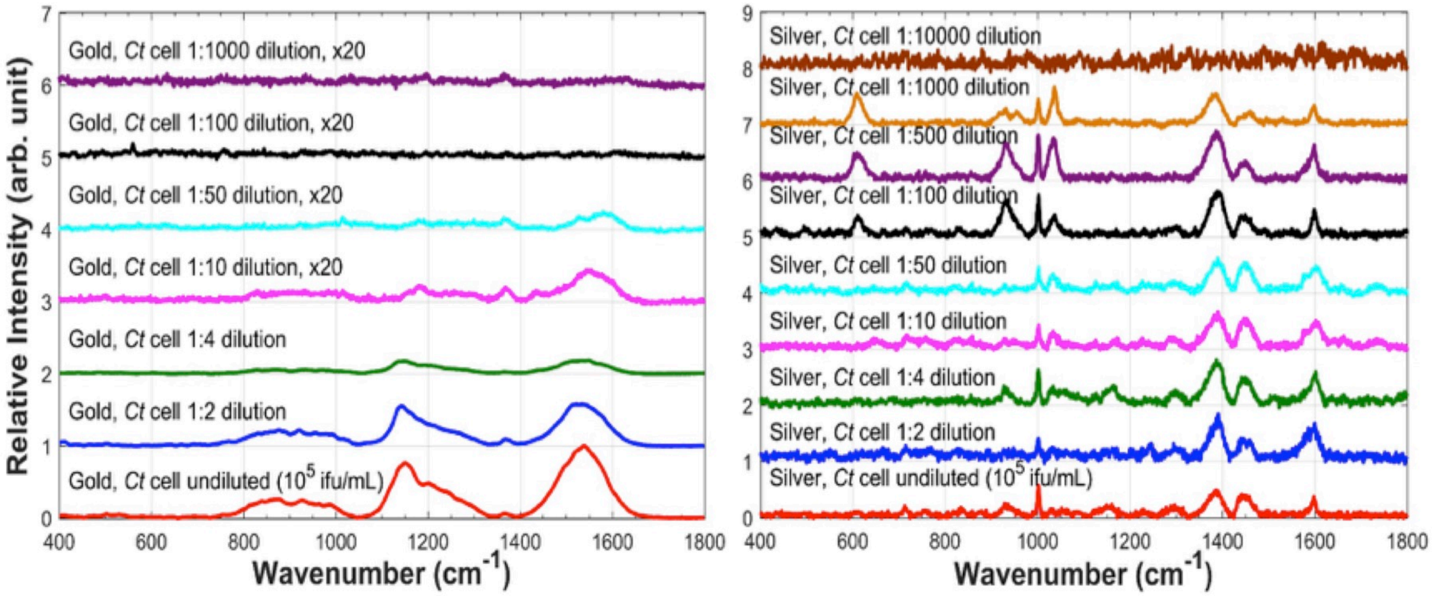

Supplementary Figure S2. Sensitivity of *C. trachomatis* (*Ct*) on Au and Ag SERS substrate at t = 0 min (right after sample processing). The initial concentration of elementary bodies is determined by titer to be ~10^5 ifu/mL. The lowest concentration of EBs to yield SERS signal on Au was ~10^4 ifu/mL and ~10^2 ifu/mL on Ag by the simple centrifugation enrichment procedure employed here.

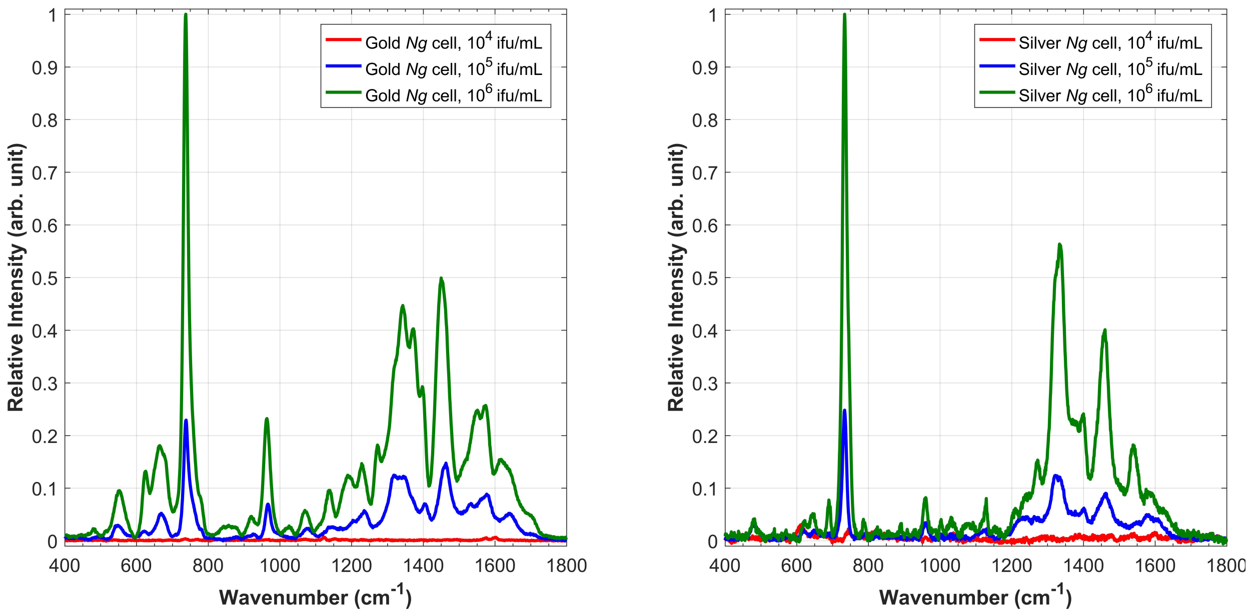

Supplementary Figure S3. Sensitivity of *N. gonorrhoeae* (*Ng*) on Au and Ag SERS substrates at t = 60 minutes post sample processing. The initial concentration of *Ng* is determined by serial dilution and overnight cell culture to be ~ 10^7 cfu/mL. The lowest concentration of bacterial cell to yield SERS signal is ~ 10^5 cfu/mL on both metals.

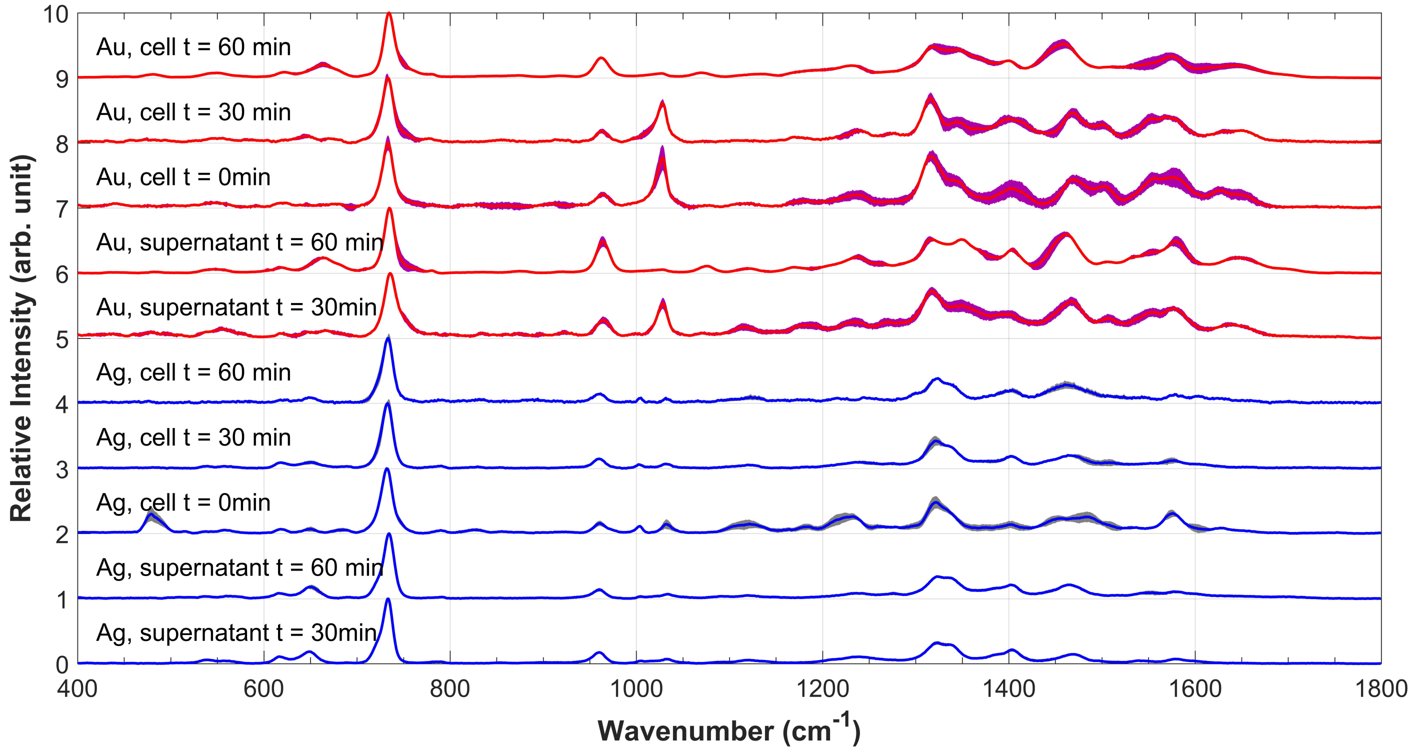

Supplementary Figure S4. Standard deviation of the SERS spectra for multiple growths of *N. gonorrhoeae* are plotted on top of the SERS spectra shown in Figs. 2 - 4 for Au (red) and Ag (blue) substrates. The normalized averaged spectra (~10) of 4 and 2 independent bacterial growths contribute to the plotted standard deviation spectra for the cells and supernatant of *N. gonorrhoeae* on Au. Correspondingly, averaged spectra (~10) of 3 growths contribute to the standard deviation spectra for the cells and supernatant of *N. gonorrhoeae* on Ag. The grey area represents ± σ (one standard deviation). The standard deviation spectra are plotted relative to those shown in Fig.4 demonstrated the high degree of reproducibility of these spectra at each time and on both Ag and Au. This figure highlights that the reported results are not growth dependent and are a robust signature of this bacterial strain.

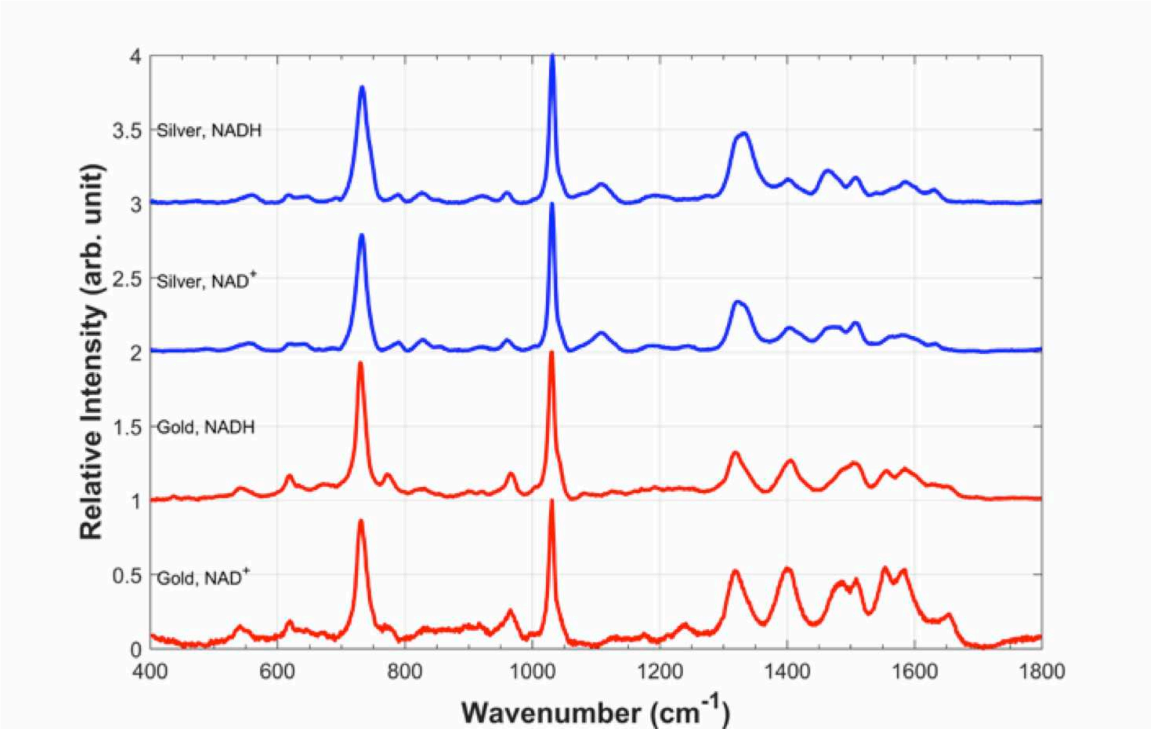

Supplementary Figure S5. SERS specta of NAD^+ and NADH on Ag and Au substrates.

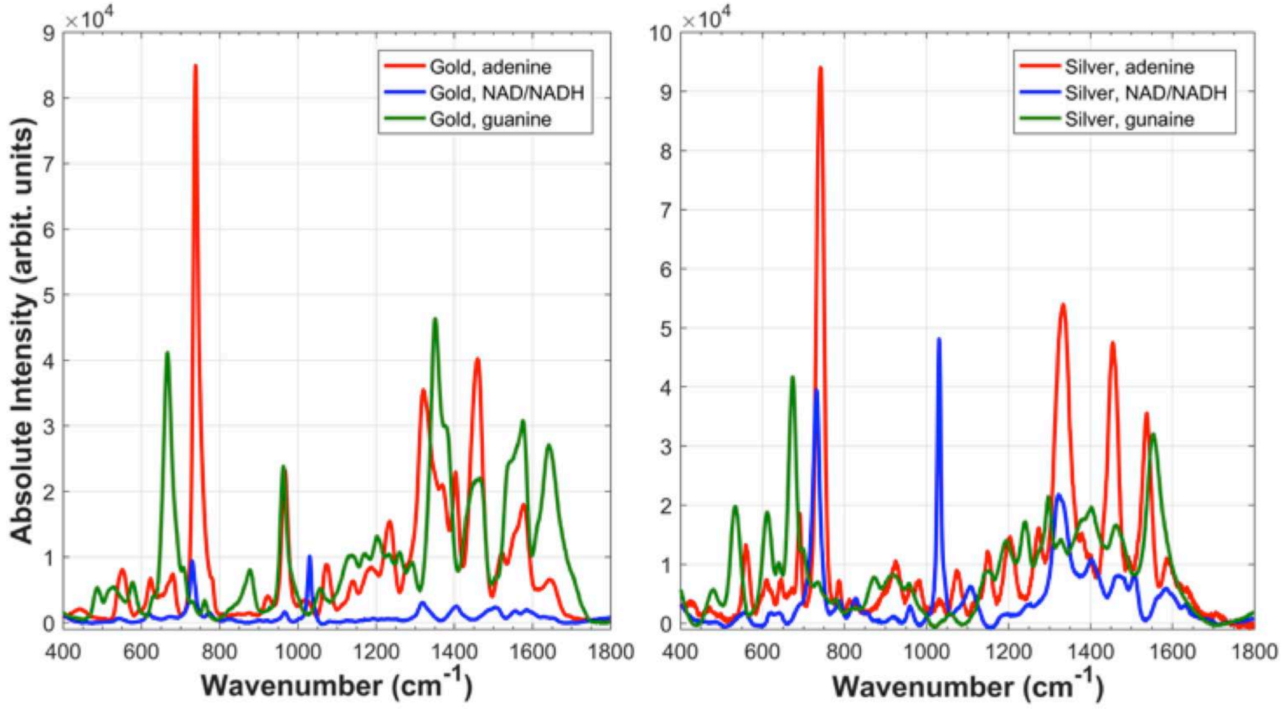

Supplementary Figure S6. Relative SERS intensities of 20 μM solutions of each major component contributing to the SERS spectrum of *N. gonorrhoeae* on Au and Ag substrates.

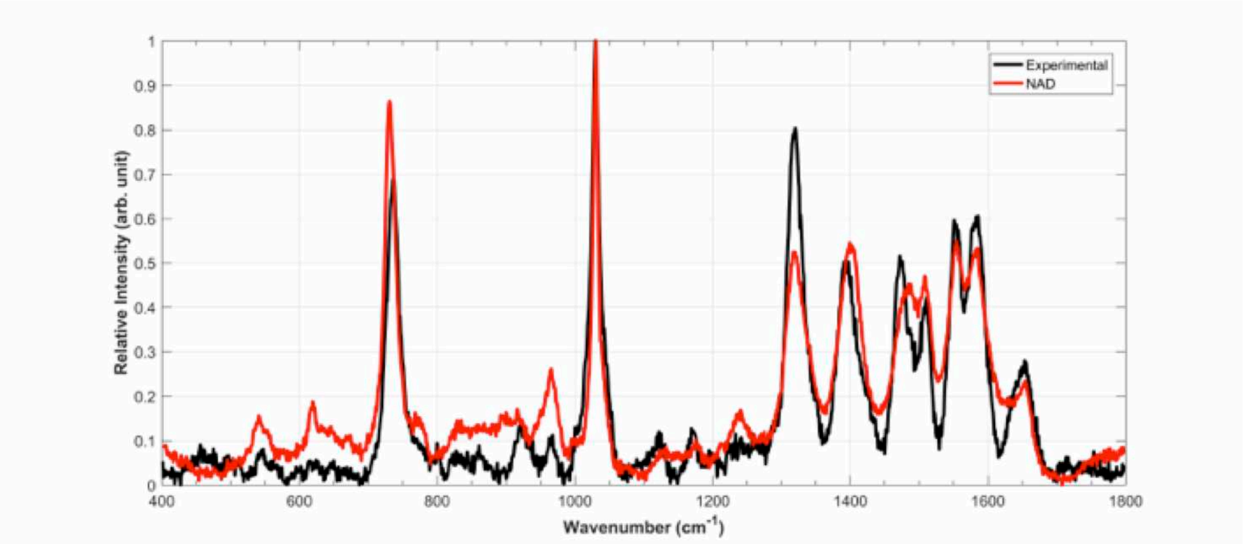

Supplementary Figure S7. SERS spectrum of *N. gonorrhoeae* on Au after 60 minutes of the intial water washing and s subsequent re-wasihng with water. All observed peaks can be assigned to NAD^+ features. More specifically virtually no adenine or guanine is observed in SERS spectrum of this 60 min rewashed sample.

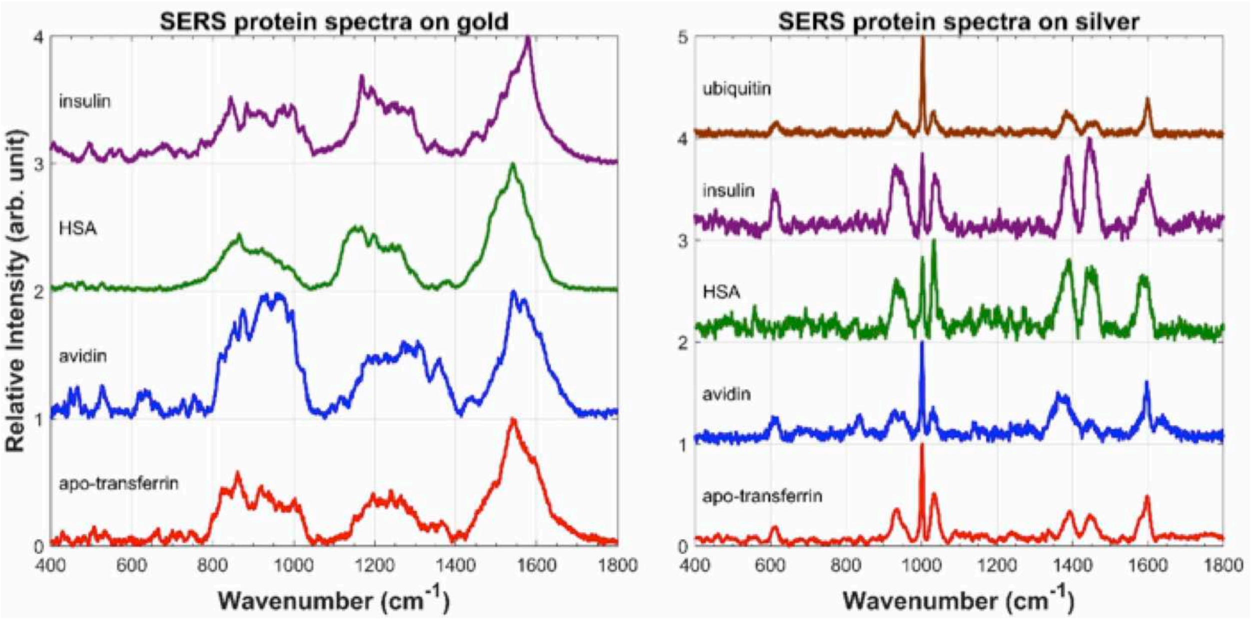

Supplementary Figure S8. SERS spectra of various protein molecule solutions in water on the gold substrate (left) and the silver substrate (right). On the gold substrate all protein molecules display three broad spectral features center around 1550 cm^{-1}, 1200 cm^{-1} and 900 cm^{-1}, but each protein can still be distinguish from each other from the relative intensity of the three broad features. On the silver substrate all protein display similar, narrower spectral bands but each protein can still be distinguished based on the difference in relative peak intensity.

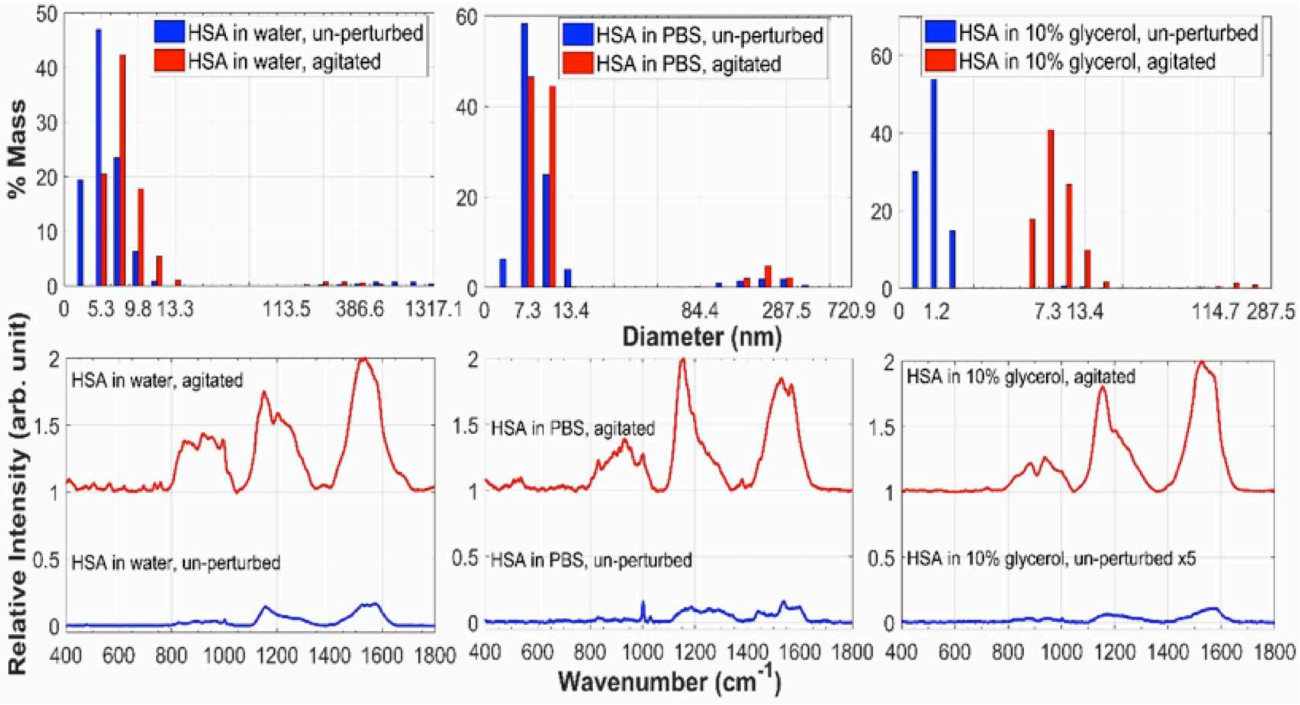

Supplementary Figure S9. Comparison of SERS spectra on Au substrates (bottom) and dynamic light scattering (DLS) measurements (top) of HSA in three different solvents; water, PBS and glycerol, before and after mechanical agitation. Unperturbed corresponds to minimal mechanical agitation of the protein solutions. Agitated corresponds to vigorous pipetting of the samples to generate both shearing stress and cavitation For each solvent, the increase in particle diameter found when the solution is more vigorously agitated is correlated with an increase of the SERS intensity of the three-broad band spectrum.

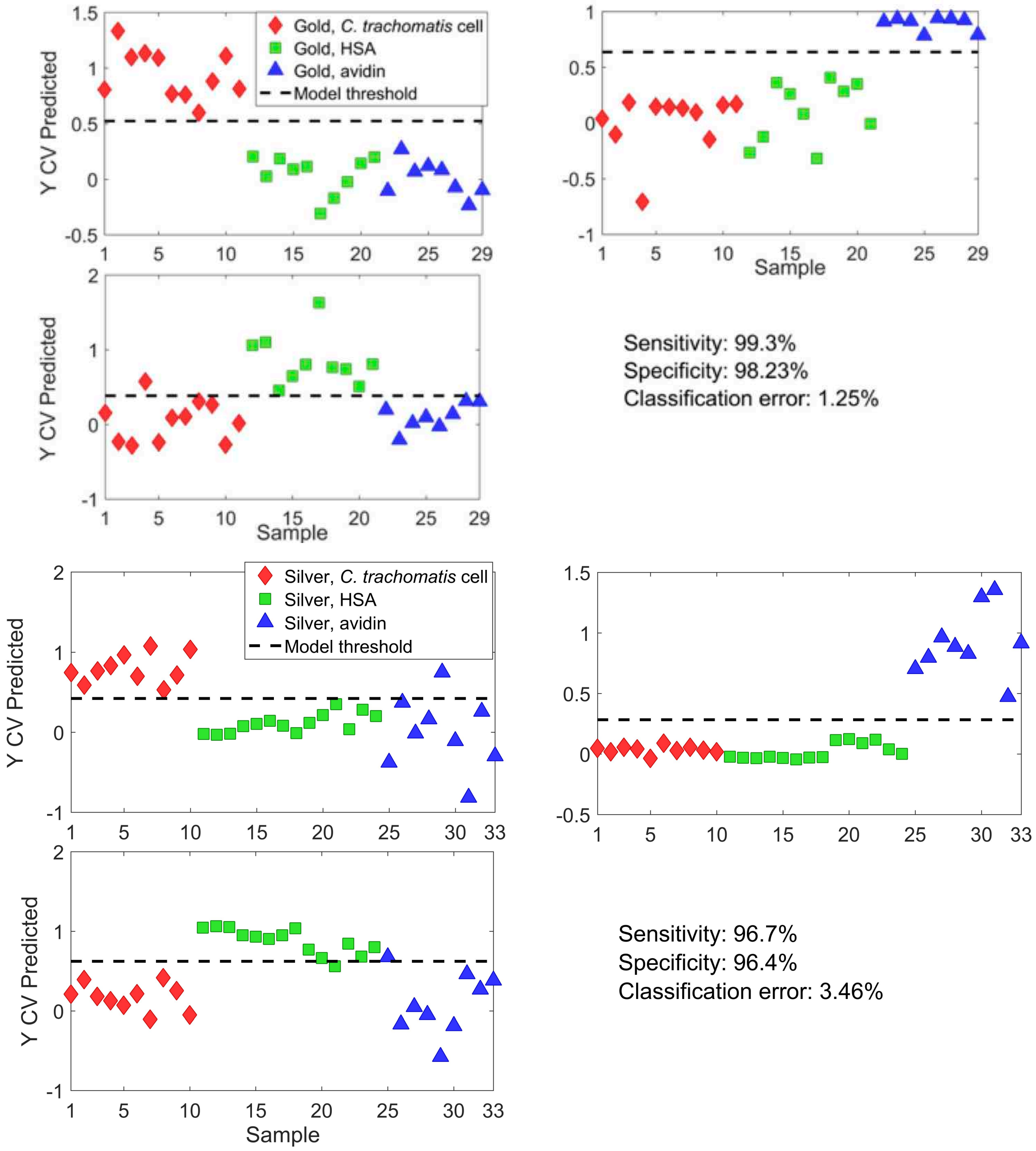

Supplementary Figure S10. Results of a PLS-DA treatment of the SERS spectra of *C. trachcomatis* and the proteins avidin and HAS (human serum albumin) on Ag and Au substrates. As seen above, this classification procedure successfully identifies these closely related spectra c with 99%/97% sensitivity and 98%/96% specificity for Au/Ag substrates.

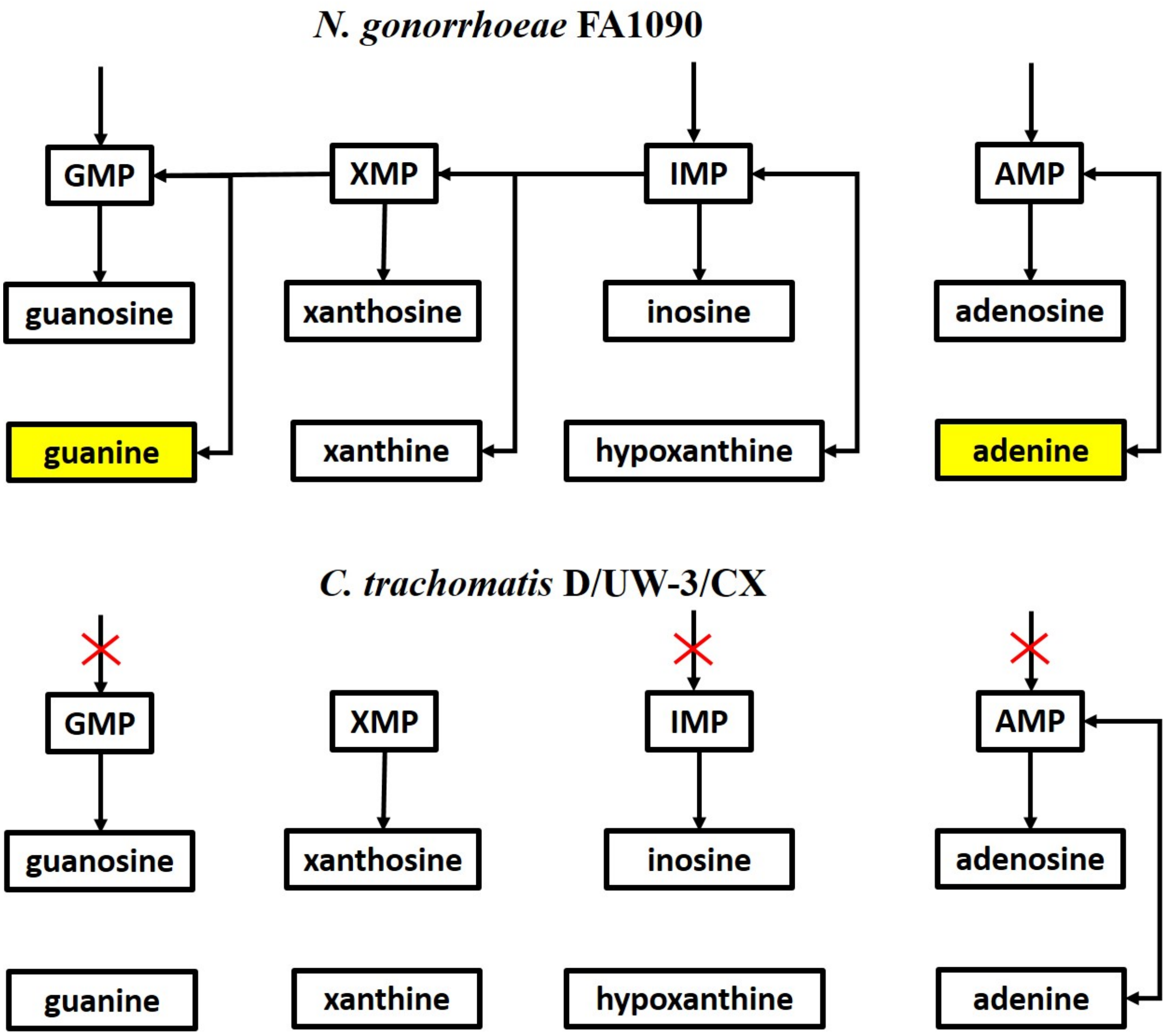

Supplementary Figure S11. A subset of the purine metabolic pathways given by the KEGG database (http://www.genome.jp/kegg/pathway/map/map00230.html) for *N. gonorrhoeae* (upper panel) and *C. trachomatis* (lower panel). Enzymatic pathways that result in the formation of the free nucleobases: adenine and guanine, are evident for *N. gonorrhoeae* but not *C. trachomatis* as seen in the reported SERS spectra. Arrows correspond to enzymes present for each of the indicated reactions.

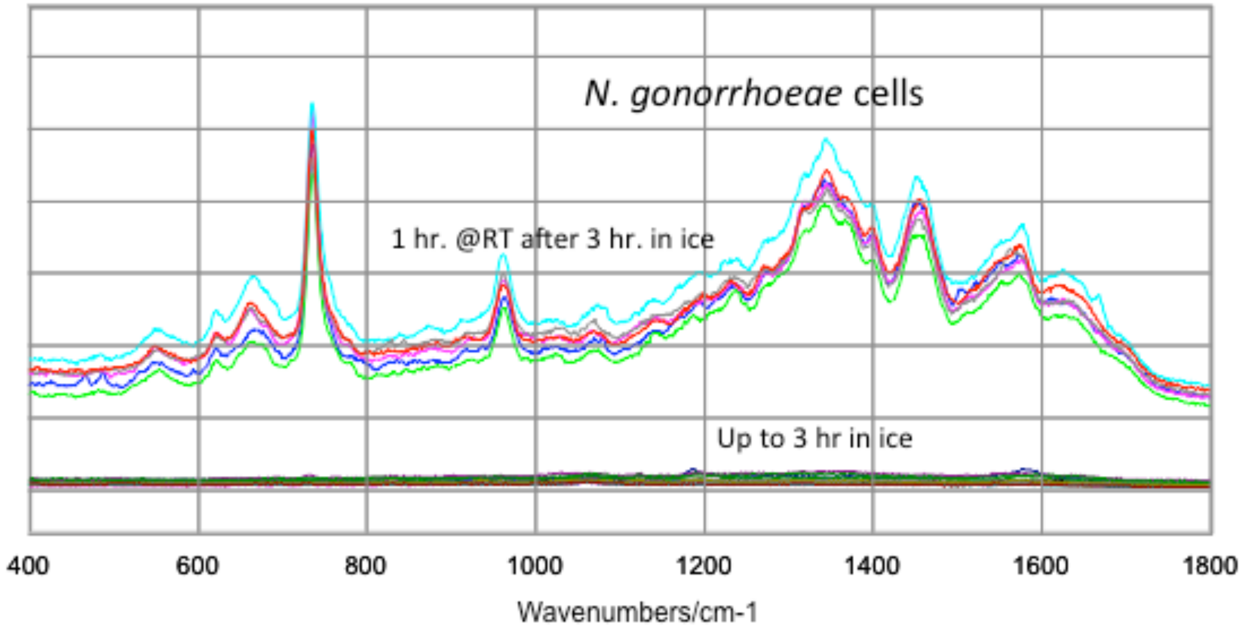

Supplementary Figure S12. SERS spectra of *N. gonorrhoeae* cells that have been held at near 0 ^{o}C compared to spectra once the same sample has been slowing brought to room temperature. The "cold" cells show no signal; once this sample warms to room temperature the metabolic purine products become evident in the SERS spectra.

Supplementary information: *in vitro* cultivation and harvest of *C. trachomatis* procedure

C. trachomatis Serovar D (VR-885) is obtained from American Type Culture Collection (ATCC®, Manassas, VA). The host cell is McCoy cell line (ATCC® CRL-1696). The host cells are grown in a 75 cm^2 Corning® cell culture flask (Corning®) with minimal essential medium (MEM, Life Technologies®) containing 10% fetal bovine serum (ATCC®) in a humidified incubator at 37 °C and 5% CO_2 until 90%-100% confluent. After infecting the host cell a special medium with the following ingredients is used for maintaining the infected host cell: 90 mL Dulbecco's minimal essential medium (DMEM, Life Technologies®), 10 mL fetal bovine serum (ATCC®) and 1 μg/mL cycloheximide (Sigma®). Cycloheximide, a known eukaryotic cell protein synthesis inhibitor, limits host cell growth thus allowing nutrients to be directed to the proliferating bacteria.

C. trachomatis cultivation is described in great detail in ATCC® cell culture guidelines[i] and in the literature.[ii] The procedure used to grow the *C. trachomatis* for these experiment follows here. The growth medium is first removed from the cell culture flask and the confluent host cell layer is treated with 45 μg/mL DEAE-dextran (Sigma®) in Hank's Balanced Salt Solution (HBSS, Life Technologies®) for 15 minutes. Then the DEAE-dextran solution is removed and the host cell layer is rinsed twice with HBSS. Then 7 mL of the special medium described above is added to the cell culture flask. A *C. trachomatis* stock sample is removed from -80 °C storage and quickly thawed (<30 seconds) in 37 °C warm water bath. The stock sample is added to the cell culture flask containing the special medium immediately after thawing. Then the cell culture flask is shaken on a platform rocker (VWR®) for three hours at room temperature. After three hours more special medium is added to the cell culture flask to bring the final volume to 10 mL. The flask is labeled and incubated at 37 °C with 5% CO_2 and humidity for 72-96 hours with daily monitor by visual examination under inverted microscope for the growth and the size change of the inclusion bodies.

The following procedure is carried out in order to harvest the *C. trachomatis* cells. After 72-96 hours of incubation, the host cells are lysed by disruption with sterile glass beads. The contents are immediately transferred to a sterile 50 mL conical centrifuge tube (Corning®) and stored in ice. The cell culture flask and the glass beads are rinsed twice with 10 mL of ice-cold HBSS and added to the 50 mL conical centrifuge tube. The conical tube is centrifuged at x1000g at 4 °C for 15 minutes to remove cell debris. The clear supernatant is transferred to a sterile 30

mL high-speed round-bottom PPCO centrifuge tube (Nalgene®) and centrifuge at x30, 000g at 4 °C for 30 minutes (Sorvall® RC-5C plus). After centrifugation the supernatant is discarded and the pellet at the bottom is rinsed twice with 2 mL of ice-cold HBSS. Then the pellet is re-suspended in 300 µL of ice-cold HBSS and aliquot into three 0.5 mL microcentrifuge tubes and stored at -80 °C.

To determine the quantity of chlamydiae in the frozen sample, one McCoy cell frozen sample (~10^5 cell/mL) is divided into half and used to seed two 75 cm^2 cell culture flasks (Corning®). The cells in both flasks are grow to 100% confluent. One frozen chlamydiae stock sample (100 µL) is used to infect one of the flasks. The McCoy cells in another flask are removed and the total number of cells in the flask are counted with a hemocytometer. After 24 hours the infected cells are viewed under the microscope with 40x objective. The number of inclusion bodies and the total number of host cell in each view are recorded. The numbers from ten views are averaged and the ratio of inclusion bodies to total number of host cells are calculated. Since it's assumed that the total number of host cells in both cell culture flask is the same with growth to 100% confluent, the number of chlamydiae inclusion forming unit (ifu/mL) can be determined by multiplying the ratio of inclusion bodies/total number of host cell with the total number of host cells. With this method, the number of chlamydiae in the 100 µL frozen sample is determined to be ~10^5 ifu/mL.

Supplementary information: *in vitro* cultivation and harvest of *N. gonorrhoeae* procedure

N. gonorrhoeae (FA1090) was obtained from ATCC®. This bacterial strain is grown in 814 broth medium supplemented with bovine hemoglobin and IsoVitaleX® (BD®). The agar medium for this bacteria is chocolate II agar (GC II agar) with IsoVitaleX® supplement which is commercially available (BD®). The 814 broth medium is prepared according to ATCC® receipt[iii] and sterilized at 121 °C after preparation. However, we found that the hemoglobin in the broth medium aggregates and precipitated out of the broth medium after sterilization. When using this broth medium to grow the bacteria, it's almost impossible to completely separate the bacterial cells from the hemoglobin aggregation by centrifugation. To solve this problem we grew the bacteria on the GC II agar and harvest them by stripping them off the plate with a sterilized loop. We developed a reliable procedure to yield log-phase *N. gonorrhoeae* that produced robust signals on our SERS substrates using combinations of the broth medium and the

GC II agar. First the bacteria is grown on a GC II plate from frozen stock sample overnight at 37 °C with 5% CO_2. Then three to four colonies from the plate are removed with a sterilized loop and inoculated into a sterile flask containing 20 mL broth medium. The bacteria are allowed to grow in the broth medium with constant shaking for 20 hours at 37 °C and 5% CO_2. After that, one loop (~ 10 μL) is used to inoculate a fresh GC II agar plate and incubated for 18 hours at 37 °C and 5% CO_2. The bacteria cell are harvested from the agar plate by stripping the colonies off the plate with sterilized loop.

---

[i] ATCC® product information for *Chlamydia trachomatis* Serovar D, https://www.atcc.org/products/all/VR-885.aspx

[ii] Scidmore MA (2005) *Curr. Protoc. Microbiol.* Chapter 11:Unit 11A 11.

[iii] ATCC® product information for *Neisseria gonorrhoeae* FA1090, https://www.atcc.org/~/media/7CD43F06265B4AD382D91947F95DF380.ashx
